# Using soil bacterial communities to predict physico-chemical variables and soil quality

**DOI:** 10.1186/s40168-020-00858-1

**Published:** 2020-06-02

**Authors:** Syrie M. Hermans, Hannah L. Buckley, Bradley S. Case, Fiona Curran-Cournane, Matthew Taylor, Gavin Lear

**Affiliations:** 1 School of Biological Sciences, University of Auckland, 3A Symonds Street, Auckland, 1010 New Zealand; 2 School of Science, Auckland University of Technology, 46 Wakefield St, Auckland, 1010 New Zealand; 3 Ministry for the Environment – Manatū Mō Te Taiao, 45 Queen Street, Auckland, 1010 New Zealand; 4 Waikato Regional Council, 401 Grey Street, Hamilton, 3216 New Zealand

**Keywords:** Bacterial communities, Bacterial indicators, Biomonitoring, Environmental monitoring, Random forest analysis, Soil health, Soil microbiology

## Abstract

**Background:**

Soil ecosystems consist of complex interactions between biological communities and physico-chemical variables, all of which contribute to the overall quality of soils. Despite this, changes in bacterial communities are ignored by most soil monitoring programs, which are crucial to ensure the sustainability of land management practices. We applied 16S rRNA gene sequencing to determine the bacterial community composition of over 3000 soil samples from 606 sites in New Zealand. Sites were classified as indigenous forests, exotic forest plantations, horticulture, or pastoral grasslands; soil physico-chemical variables related to soil quality were also collected. The composition of soil bacterial communities was then used to predict the land use and soil physico-chemical variables of each site.

**Results:**

Soil bacterial community composition was strongly linked to land use, to the extent where it could correctly determine the type of land use with 85% accuracy. Despite the inherent variation introduced by sampling across ~ 1300 km distance gradient, the bacterial communities could also be used to differentiate sites grouped by key physico-chemical properties with up to 83% accuracy. Further, individual soil variables such as soil pH, nutrient concentrations and bulk density could be predicted; the correlations between predicted and true values ranged from weak (*R*^2^ value = 0.35) to strong (*R*^2^ value = 0.79). These predictions were accurate enough to allow bacterial communities to assign the correct soil quality scores with 50–95% accuracy.

**Conclusions:**

The inclusion of biological information when monitoring soil quality is crucial if we wish to gain a better, more accurate understanding of how land management impacts the soil ecosystem. We have shown that soil bacterial communities can provide biologically relevant insights on the impacts of land use on soil ecosystems. Furthermore, their ability to indicate changes in individual soil parameters shows that analysing bacterial DNA data can be used to screen soil quality.

Video Abstract

## Background

Soil quality is underpinned by a complex suite of belowground processes in both natural and agricultural ecosystems. Soil quality is defined as the ability of soil to function as an ecosystem component capable of maintaining the quality of surrounding air and water while supporting plant and animal productivity [1]. High-quality soils are therefore crucial for sustaining agricultural and pastoral industries upon which both food security and financial stability depend [2]. Soils harbour a rich collection of microbial life [3], which contribute to the cycling of important nutrients [4], impact plant growth [5] and can act as, or protect other organisms from, pathogens [6]. Macroorganisms interact with microorganisms to facilitate this and independently are important for processes such as decomposition [7]. Despite the importance of living organisms for maintaining healthy soil ecosystems, most initiatives that directly monitor soil quality for applied purposes focus on changes in abiotic variables such as soil nutrients, metal pollutants and soil structure [8]. Where biological measures are included in monitoring efforts, they are often crude and generalized, such as microbial biomass or soil respiration [8], although some use more specific organisms, such as earthworms, as more sensitive indicators [9]. As well as relaying important information about the biological functioning of the ecosystem, soil organisms only respond to bioavailable nutrients and contaminants, unlike chemical measures which reflect the total proportion present [10]. Better incorporation of biological indicators in soil monitoring will provide a more sensitive, relevant and holistic insight into how anthropogenic activity impacts the soil environment.

Soil bacterial communities are strongly impacted by changes in soil conditions. The diversity and composition of bacterial communities change with changing soil acidity [11–13]. At national scales or larger, this is often observed to be the strongest explanatory variable for bacterial community richness [14, 15] to the extent where large-scale predictions of bacterial diversity are possible based on pH data alone [16]. Additionally, plant diversity, nutrient concentrations, soil moisture and soil type have all been shown to correlate with changes in bacterial communities [14, 17, 18]. Importantly, there is ample evidence that bacterial communities directly, or indirectly, respond to changes in the soil environment brought on by anthropogenic activity. Land use has been shown to correlate with changes in bacterial community composition [19], and heavily managed soils contain distinct bacterial communities compared to unmanaged soils [20]. More specifically, management practices such as fertilising, altering soil pH and creating monocultures of plants or animals have all been shown to influence soil microbial communities [21–23]. Overall, the composition of bacterial communities appears to be heavily influenced by changes in the soil environment, many of which are the direct result of land use activities.

Given their ubiquitous nature, and sensitivity to environmental changes, bacterial communities are gaining recognition as useful indicators of environmental health [24]. In stream ecosystems, bacterial communities have been shown capable of indicating the level of catchment disturbance, with results correlating with both abiotic water quality data and traditional macroinvertebrate community indicator data [25]. In soil ecosystems, similarly strong correlations between specific microbial taxa and soil variables have been reported, suggesting microbial community data can be used to indicate changes in physico-chemical conditions [17], serve as indicators of ecological restoration [26] and even predict crop yields [27]. While progress has been made towards better understanding how bacterial communities can be indicative of environmental health, more effort needs to be made, and soil bacteria remain largely understudied in this regard. Investigating if soil bacterial communities respond in a predictable manner to human land use and soil physico-chemical changes across a wide variety of different soils, spatial gradients and climatic conditions will reveal their potential to serve widely as indicators of soil quality.

There are many statistical methods available for indicator development based on bacterial community data; particularly promising are machine learning approaches [24]. Broadly speaking, these involve creating a predictive model through identifying discriminating independent variables; if successful, the model can then be used to classify new samples from an assessment of the biological data. Random forest analysis is an example of machine learning where an ensemble of decision trees are generated to iteratively identify the optimal set of explanatory variables to predict variation in a response variable [28]. Random forest models based on bacterial community composition have been successfully used to determine whether groundwater samples are contaminated with uranium or nitrate and to quantitatively predict a wide range of geochemical variables such as pH and metal concentrations [29]; similar outcomes have also been reported from the assessment of aquatic communities [30, 31]. Random forest models may outperform other modelling methods when using microbial data to predict environmental changes [29] and offer a straightforward and well-documented approach for creating predictive tools.

While there is ample evidence that soil bacterial communities act as useful indicators of soil quality, there is a lack of research directly exploring this. Incorporating biologically relevant measures of soil quality is essential for efficiently monitoring whether agricultural and pastoral practices are conducted in a sustainable manner. Therefore, using an extensive dataset of soil samples collected from a variety of different natural and managed land uses across New Zealand, we aimed to (1) determine how bacterial communities in managed soils differ to those in natural, undisturbed environments, (2) determine the extent to which bacterial communities in managed soils can predict soil physico-chemical characteristics and (3) explore if these predictions are accurate and reliable enough to be applied for soil quality monitoring.

## Results

**Fig. 1:**
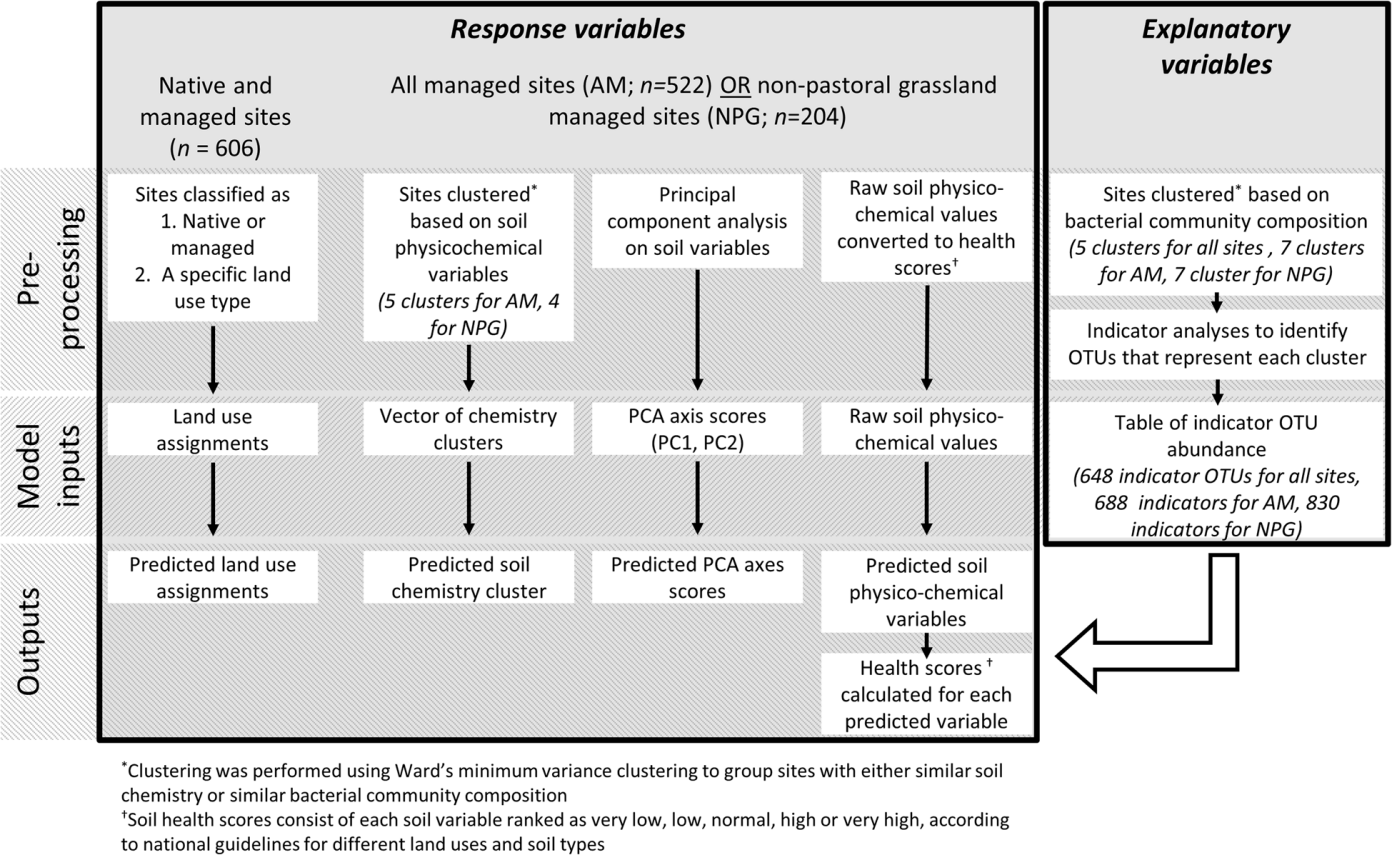
Summary of the steps taken to produce the random forest models. A range of models were created, based on three different subsets of the data: all native and managed sites, all managed (AM) sites only, or non-pastoral grassland (NPG) managed sites only. Random forest analyses were performed using the ‘randomForest’ package with default parameters (Liaw and Wiener 2002)

The composition of soil bacterial communities was determined for 606 sites across New Zealand (Fig. S1, Additional file 1). These sites were categorized as being dominated by indigenous forest, exotic forest, horticulture or pastoral grasslands; soil physico-chemical variables were collected to characterise the soil environment. Random forest models were then used to assess if bacterial community composition could be used to predict the land use type, general soil characteristics and specific soil physico-chemical variables (Fig. 1).

### Soil bacterial community composition across different to land use types

**Fig. 2:**
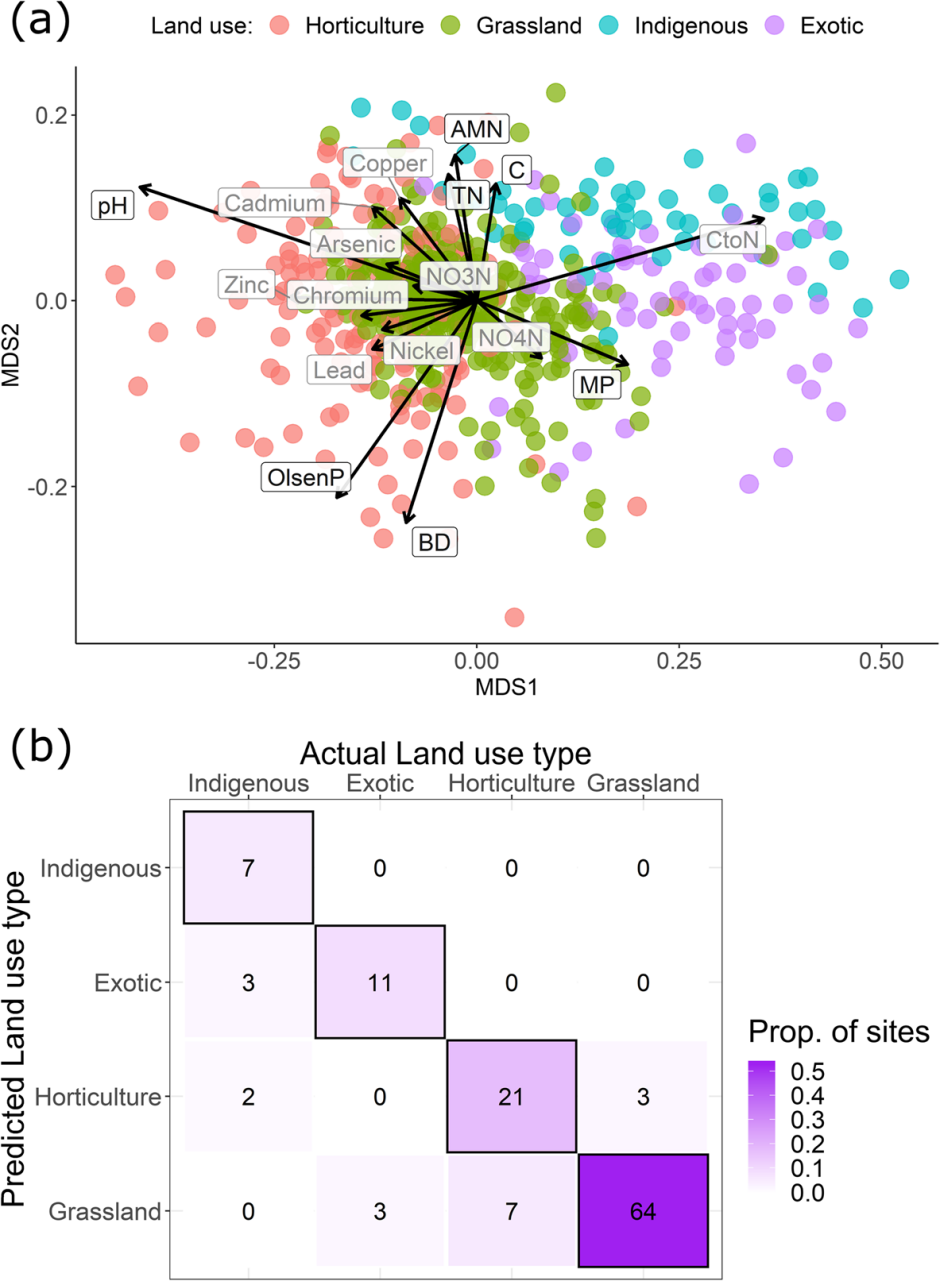
**a** Relative compositional differences (Bray-Curtis dissimilarity) between bacterial community composition at sites with different land uses. Vectors represent soil environmental variables which significantly correlated with the ordination (*P* < 0.05 based on 999 permutations); variables in black represent those with well-defined soil quality guidelines which were therefore used in subsequent modelling. Stress value for the ordination was 0.14. **b** The number of correct (*n* = 103) and incorrect (*n* = 18) predictions of land use type, based on a random forest classification of bacterial community data. Black borders indicate correct classifications

Bacterial community composition was significantly different in each of the four land uses (PERMANOVA pairwise adjusted *P* < 0.01), and land use was able to explain 17.9% of the variation in bacterial community composition. The measured soil variables correlated significantly with underlying differences in bacterial community composition among sites. Of the explanatory variables, pH and C:N had the highest correlations (Fig. 2a). C:N was higher in the two forested land uses, while horticulture sites had higher concentrations of Olsen P, and higher bulk density (Fig. 2a).

Using random forest models, we confirmed that the composition of bacterial communities was strongly linked to land use, to the extent where soil bacteria at a site could be used to predict the land use with 85% accuracy (Fig. 2b).

### Using bacterial community composition to predict soil conditions

**Fig. 3:**
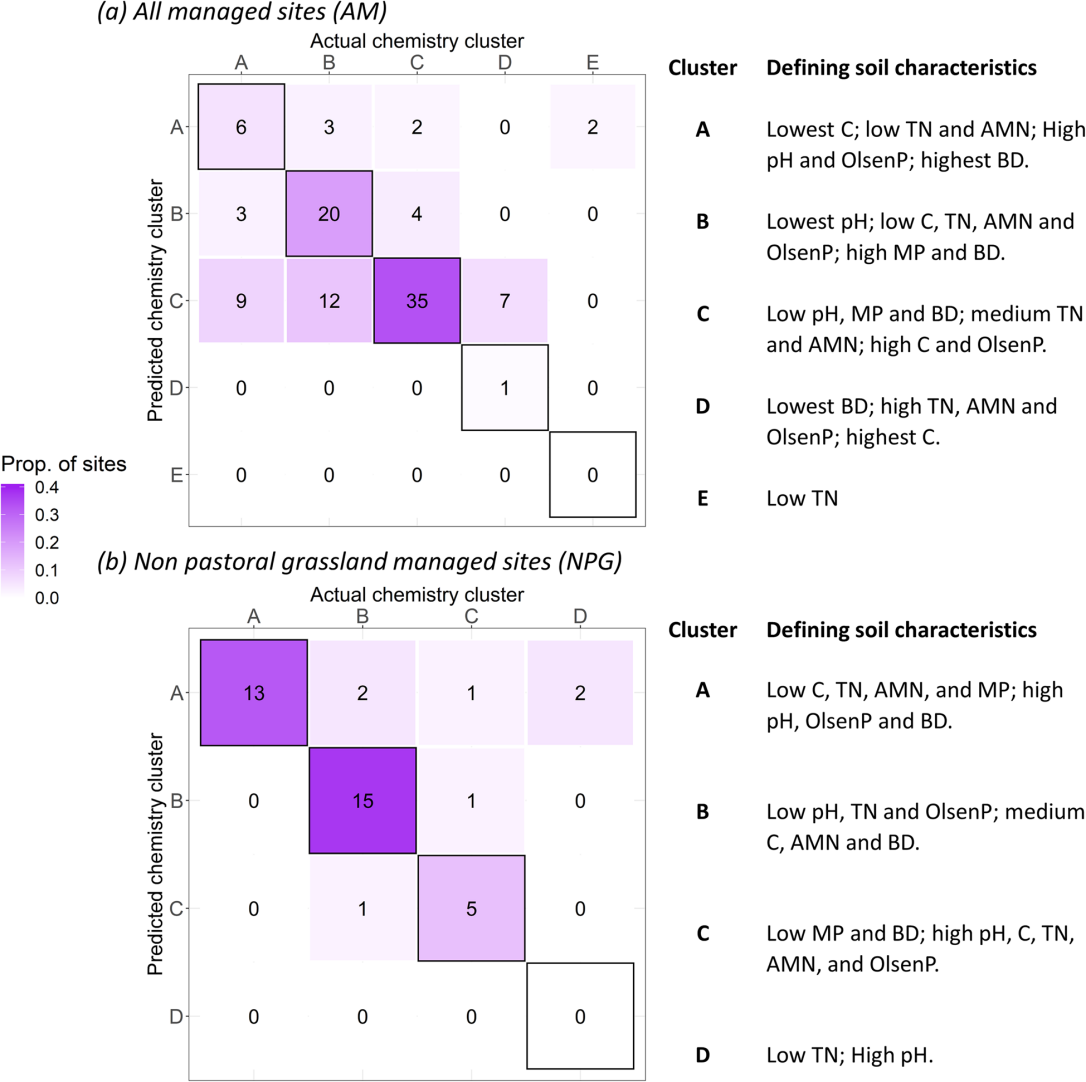
The number of correct and incorrect predictions of the chemistry cluster to which a site belongs, based on a random forest classification of bacterial community data. Models were based on either **a** all sites belonging to all managed (AM) land use type (horticulture, exotic or pastoral grassland) or **b** sites belonging to non-pastoral grassland (NPG) managed land uses. Black borders indicate correct classifications (**a***n* = 62 out of 104; **b***n* = 33 out of 40). Each cluster can be defined by the soil characteristics of the sites within those clusters, as indicated to the right of each matrix

For subsets of bacterial community data, including data from either all managed (AM), or all non-pastoral grassland managed (NPG) sites, ‘soil clusters’ were assigned for which the soil physico-chemical environment could be generally defined. For example, cluster A for the AM bacterial dataset contained sites which in general had the lowest carbon, low total nitrogen and anaerobic mineralizable nitrogen, high pH and Olsen P concentration and the highest bulk density when compared to all other sites in different clusters (Fig. 3a). Using random forest models, the cluster to which a site belonged could be correctly predicted 60% of the time for AM sites and 83% of the time for NPG sites based only on assessment of the bacterial community data. Groups E and D for the AM and NPG sites, respectively, had small sample sizes and consisted of outlier sites; this likely contributed to the fact that these clusters could not be correctly assigned. In general, incorrectly assigned sites tended to be located on the border of the data cluster when plotted based on PCA scores, whereas correct assignments were typically located closer to the centroid of their group (Fig. S2, Additional file 1).

**Fig. 4:**
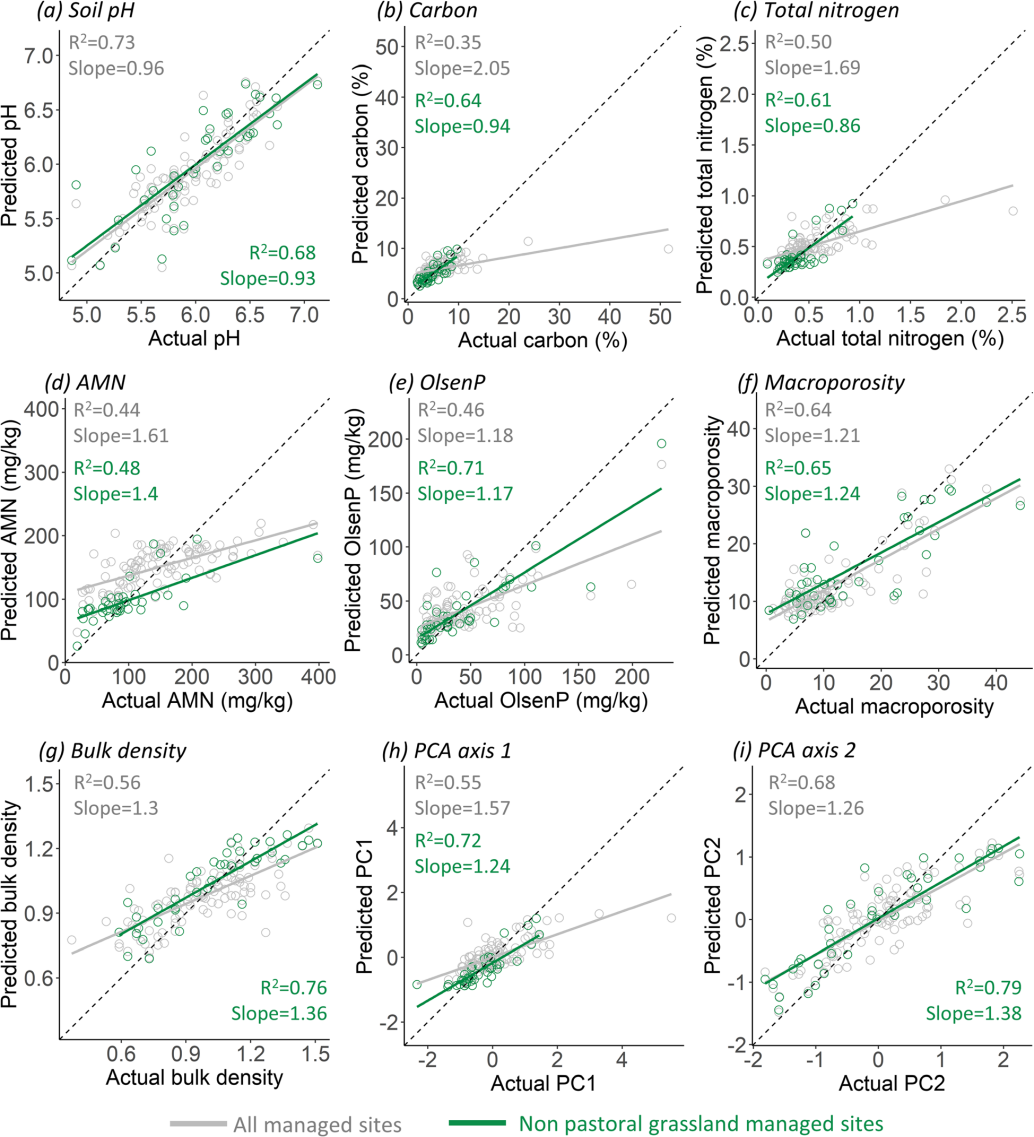
Predicted **a**–**g** soil variable values or **h**, **i** PCA axes scores based on random forest regression analyses versus actual values. Models were based on either (in grey) all sites belonging to a managed land use type (AM; horticulture, exotic or pastoral grassland) or (in green) sites belonging to non-pastoral grassland managed land uses (NPG). Dashed black lines indicate where points should fall for a perfect prediction. Adjusted *R*^2^ and slope values for each linear regression are indicated on the plots

The bacterial communities at each site were also used to predict individual soil physico-chemical variables and soil PCA scores (Fig. 4). When including all managed (AM) sites, regression models comparing the predicted to actual soil variables ranged from weak to strong correlations (adjusted *R*^2^ 0.35–0.73). Excluding the pastoral grassland sites resulted in moderate to strong correlations (adjusted *R*^2^ 0.48–0.79, Fig. 4); models containing only pastoral grassland sites performed poorly (Fig. S3–S4, Additional file 1). The pastoral grassland sites had a weaker relationship between bacterial community dissimilarity and soil environmental differences compared to other land uses (Fig. S5, Additional file 1), despite comparable variability in bacterial community composition and the soil environment (Fig. S6, Additional file 1).

Predicting pH was more accurate for AM sites (slope = 0.96) than for NPG sites (slope = 0.93). NPG models had slopes closer to 1 (which represents a perfect prediction) when predicting all variables except macroporosity, bulk density and PCA axis 2 (Fig. 4). To confirm that the success, or otherwise, of each model was not biased by the combination of selected ‘validation’ sites, 100 different randomly selected subsets were created and analysed. These results were consistent with what was found using a single subset (Fig. S7, Additional file 1).

**Fig. 5:**
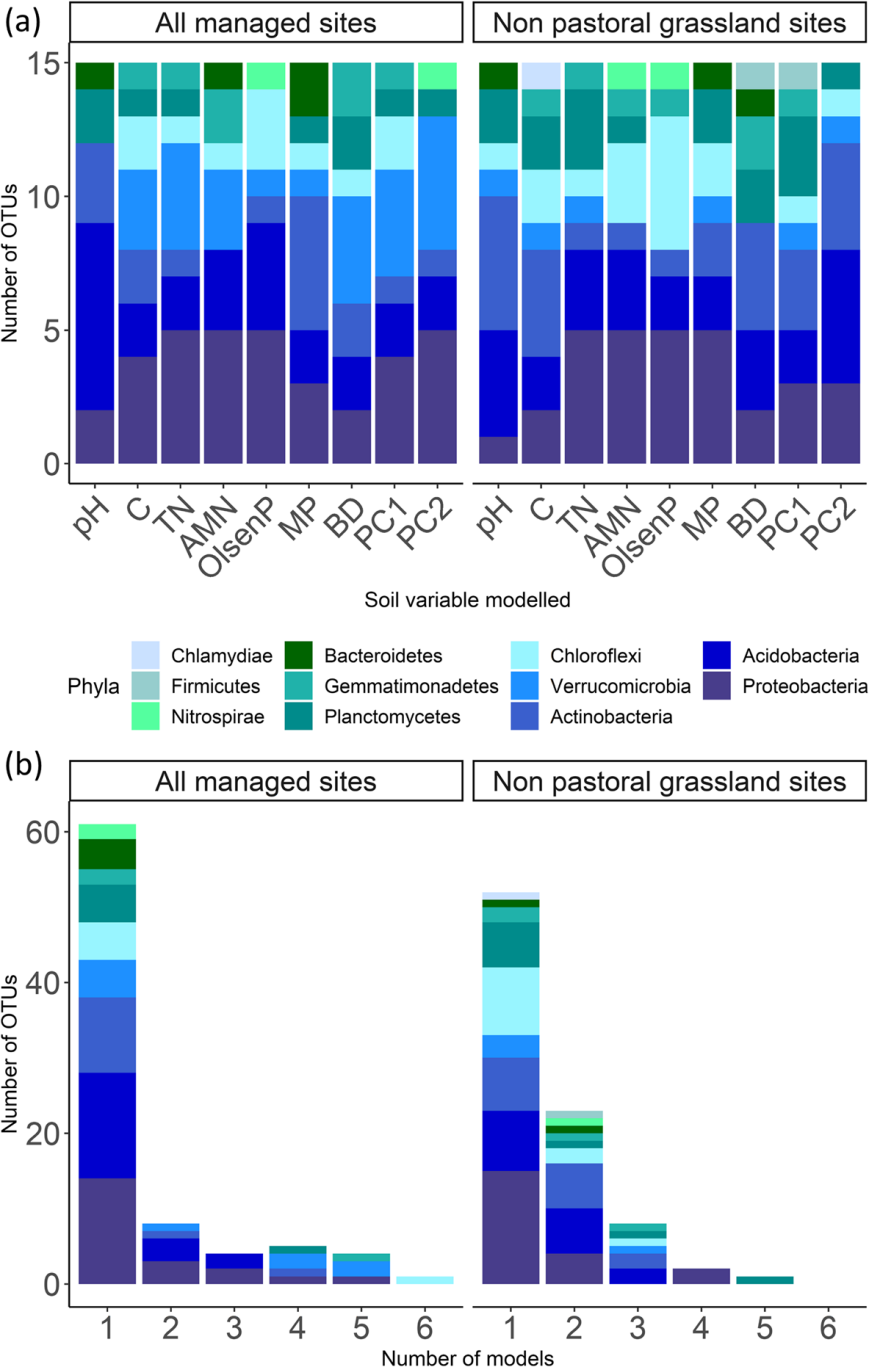
Phylum-level classification of the OTUs which comprised the top 15 most important taxa for each random forest model. **a** The models for which each OTU was important. **b** The total number of models for which each OTU was important, while there were nine models (one for each soil variable predicted), no single OTU was important in more than six models

*Proteobacteria*, *Acidobacteria* and *Actinobacteria* were the most abundant phyla across all the soil samples (Fig. S8, Additional file 1), and at least half of the OTUs which were the most important for each random forest model belonged to these phyla (based on the decrease in mean squared error when those OTUs are included in the model; Fig. 5). Several of the important OTUs for the AM models were *Verrucomicrobia*, but this taxon was less abundant amongst the important OTUs for the NPG model (Fig. 5a). Full taxonomic information for the OTUs identified as being important for the models are provided in Tables S1–2 (Additional file 1). For AM sites, the majority of the top 15 most important OTUs were unique for each model, while for NPG sites, ~ 55% of OTUs were important in at least two models (Fig. 5b).

### Determining the quality status of soils based on predicted physico-chemical values

**Fig. 6:**
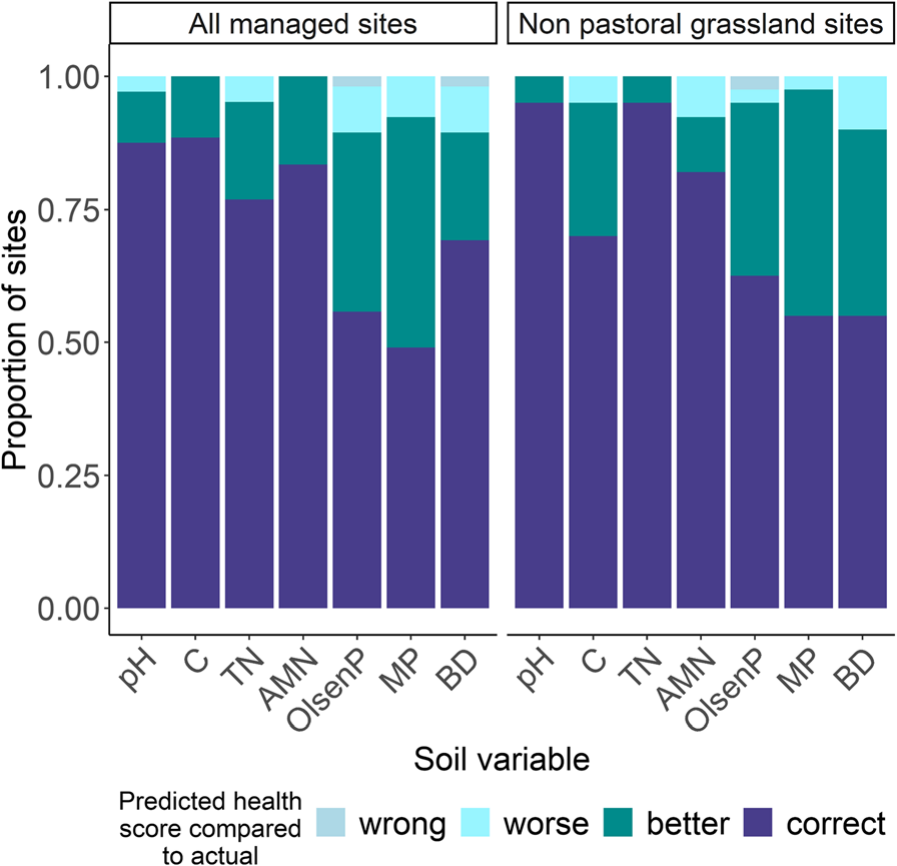
The accuracy of the soil variable quality scores calculated from the models in Fig. 4a–g. Soil quality categories for each variable were calculated while considering land use type and/or soil type. Predicted soil variables resulted in either the correct quality score (according to the quality score assigned to the actual value), a worse or better quality category, or a quality category of equal magnitude but the wrong direction (e.g. extremely high when the real score was extremely low). Detailed thresholds for each variable can be found in Tables S3-9 (Additional file 1)

Many current quality monitoring guidelines have recommended ranges for specific soil variables that are considered acceptable [32, 33]. According to these guidelines the predicted values from Fig. 4 were converted to the following categories: very low, low, normal, high and very high (see Tables S3–S9, Additional file 1 for more details). We then determined if the predicted variables resulted in the correct assignment (e.g. a site’s actual score was ‘low’ and the predicted score was also ‘low’), a better assignment (e.g. a site’s actual score was ‘low’ but the predicted score was ‘normal’) or a worse assignment (e.g. a site’s actual score was ‘low’ but the predicted score was ‘very low’). For both the models incorporating all managed (AM) sites, and models using only non-pastoral grassland managed (NPG) sites, the predicted variables were assigned to the correct categories at least 50% of the time (Fig. 6). The pH categories were predicted correctly 87.5% and 95% of the time for AM and NPG models, respectively, while the TN categories were also predicted correctly 95% of the time for the NPG model. Where the models were incorrect, they tended to result in better quality scoring categories than what was true.

## Discussion

Given the importance of maintaining a healthy and productive soil environment for sustainable global crop production, food stability and economic growth [2], improving current soil monitoring programs is highly beneficial. Here, we explored the use of soil bacterial communities as indicators of human impact, and changes in specific soil variables directly related to soil quality. Our results indicate that bacterial communities are strongly manipulated by land management practices, bacterial community data formed groups based on similar soil conditions, and specific qualitative values of soil variables could be successfully predicted. This work reveals the exciting potential of soil bacterial communities to be utilised as bioindicators of soil quality.

The presence of human activity in a site could be accurately predicted from the composition of bacterial communities, despite the inherent variation introduced by sampling across a ~ 1300 km distance gradient, with diverse soil types. This supports previous reports of the impacts human activity has on bacterial communities [19, 23]. However, there were clear weaknesses in our models, especially in the assignment of indigenous sites compared with other land uses. This could in part be due to greater similarities in bacterial communities between some indigenous and managed sites, which has been previously reported [34]. However, this weak result was most likely due to the small sample size from indigenous forest soils. The sampling strategy of the soil monitoring program that collected the samples for this study prioritises high-risk soils (i.e. those most heavily impacted by human land use) as an efficient use of monitoring resources. However, this sampling strategy inevitably leads to an underrepresentation of low-risk soils such as those in indigenous forests. The aboveground plant species composition of native forests can vary depending on the dominant canopy species and latitudinal location of the forest [35]. The relationships between aboveground plant cover and soil bacterial communities are well documented ([36, 37] [38]), and since it is likely that not enough of the variation in forest types was captured by our sample size, this could explain the reduced accuracy of the model. Indeed, most of the incorrectly assigned indigenous forest sites were in southern New Zealand, while most samples taken in indigenous forest sites were from northern New Zealand. The inclusion of data from a wider range of native forests therefore could improve the predictive power of the models.

While pastoral grassland sites were correctly classified to their land use type with the highest accuracy, the bacterial communities served as poor predictors of specific soil variables. The poor modelling results were not due to insufficient variability in bacterial communities, but rather likely reflect that the variability is related to other unmeasured variables. If bacterial communities are responding more strongly to changes or differences not related to soil quality, their ability to predict soil quality will be weaker. The differences in bacterial community composition at grazed grassland sites can be related to changes in soil variables such as pH, soil fertility and soil organic matter [39, 40]. However, there are additional factors that impact bacterial communities in pastoral grassland sites such as geographical distance, climate and the intensity of grazing [17, 41]. Measuring, and accounting for these additional sources of variation may improve the models based on pastoral grassland sites.

Soil pH, which is arguably one of the best described, and most strongly correlated variables when it comes to changes in soil microbial communities [11, 12, 16], was the most accurately predicted variable. Bacterial community composition has previously been used to accurately predict the pH of contaminated groundwater [29], and the results presented here confirm that soil microbial communities can be used in a similar manner. Predicted Olsen P, macroporosity and soil bulk values all showed strong correlations to the measured variables. These soil variables have all been previously shown to result in changes in microbial communities; increasing Olsen P by using fertilisers has been shown to result in changes in the composition and diversity of microbial communities [42], and soil compaction, indicated by decreased macroporosity and increased bulk density, has previously been identified as having a significant effect on bacterial communities [43, 44]. Anaerobically mineralizable nitrogen (AMN) did not model well, as indicated by the weak correlation between predicted and measured values. AMN has previously been shown to correlate with differences in bacterial communities [17] and indeed in our dataset correlated with bacterial community composition. The weak correlation could therefore suggest that the subset of bacterial taxa which were used in our models were not strongly related to changes in AMN.

Despite a degree of error in the predicted soil variable values, the predicted values translated to the correct ‘quality score’ much of the time, highlighting the potential of bacterial communities as indicators of soil quality. Where the quality score was incorrectly predicted, the models tended to assign a better quality score than what was true. Therefore, samples classified as having poor soil quality are likely to be reliable, while a portion of the samples our models assigned as having good quality soil using will be incorrect. This may indicate the need for further refinements, which could be achieved through the inclusion of more samples with a wider range of soil chemistries; the inclusion of a larger number of degraded sites would be especially useful as these were underrepresented in the current dataset. However, the inaccuracy of the models could also suggest that current thresholds for what is considered acceptable may need to be revisited. A major benefit of using soil bacterial communities as indicators is that they respond only to the bioavailable portions of the nutrients and contaminants in their environment, which can be greatly impacted by many soil characteristics [10]. The fact that the bacterial data did not always classify a site as outside of target ranges when the chemical data would may therefore indicate that changes in the soil chemistry are not always affecting the biological communities in the same way. This is crucial information and highlights the advantages of assessing soil bacterial communities when monitoring soil quality. Indeed, the guidelines for measuring soil quality continue to be updated [45], and the results presented here can be used to help establish new guidelines and are flexible enough to be adapted if new guidelines arise. Ultimately, we would like to use bacterial communities to add biologically relevant information, rather than as direct proxies for soil physico-chemical variables. Our results show that bacterial communities respond in a predictable manner to changes directly related to land use activities, an important first step.

The results presented here highlight the potential of bacterial communities to serve as useful indicators of soil quality; this proof of concept should encourage further research to refine and complement the findings presented here. For example, determining community composition using RNA, rather than DNA, and therefore examining the active portions of bacterial communities could highlight stronger relationships between soil quality and bacterial communities; there are examples of DNA- and RNA-based methods being used in complement to increase our understanding of microbial responses to contamination events [46]. Alternatively, exploring the bacterial communities’ functional contributions to the soil may provide information on ecosystem processes occurring within the soil and how these are being influenced by land management. Microbial genes such as those involved in the nitrogen cycle have previously been targeted to offer insights into the ecosystem services provided by bacteria [47]. Metagenomic, transcriptomic and proteomic methods are all rapidly advancing and becoming more accessible and affordable [48]. Since they provide functional insights, these methods may even increase our understanding of what constitutes a ‘healthy’ soil if we can differentiate between beneficial or negative ecosystem processes. Understanding the functional contributions of soil bacterial communities to the soil ecosystem may also allow us to better predict how our soils will function into the future as the climate and intensity of human land use continues to change. Finally, expanding the results presented here to incorporate a wider range of non-bacterial taxa that are important to soil ecosystem [49], such as fungi and other microeukaryotes, could benefit not only our ability to predict soil quality but also our understanding of the biological roles different organisms have in determining soil quality. Overall, applying additional methods to delineate the microbial communities in healthy and degraded soils has the potential for increasing our understanding of how human activity impacts the ecosystem services being provided by soil microbes.

## Conclusions

With global estimates that over a third of soil is in a state of degradation [50] increased monitoring coupled with better land management is crucial to ensure the sustainability of agricultural and pastoral industries. Given the importance of biological communities to ensure the functioning of a healthy soil ecosystem, it is time that monitoring efforts better account for changes in biotic variables, instead of relying on abiotic changes to determine the quality of soils. The research presented here shows the great potential of bacterial communities to measure the impact of human land use, and the changes these impacts have on the soil environment both generally and for specific soil variables. A greater use of the soil microbial communities as indicators in production landscapes will not only improve our ability to manage our soil resources but also contribute important insights to our understanding of what exactly constitutes ‘healthy’ soil.

## Methods

### Sample collection

Samples were collected from ten regions across New Zealand, covering approximately 196,000 km^2^ of land (Fig. S1, Additional file 1). Sample collection occurred between 2013 and 2018, and a total of 606 sites were sampled. Sites were chosen according to national guidelines [32, 51] based on the area extent of the soils and land uses. The land uses sampled included indigenous forest (*n* = 61), exotic forest (predominantly *Pinus radiata* plantation; *n* = 72), horticulture (*n* = 139) and pastoral grassland (predominantly dairy, sheep or beef farms, *n* = 334).

Sampling for molecular analyses involved collecting five individual soil cores (0–10 cm in depth, 2.5 cm in diameter) at each site across a transect at 10-m intervals. When present, leaf litter and plant biomass were displaced prior to collecting bulk soil samples. Soil samples were stored on ice until they could be transferred to − 20 °C storage at the end of the sampling day. Additional composited soil samples consisting of 25 cores collected every 2 m along the same transect were collected for soil chemical analyses, and three intact soil cores (0–10 cm deep, 10 cm wide) were collected at 15-m intervals for soil physical analyses (Table 1 [32];).

**Table 1:** Metadata collected at each site. While a range of soil variables were collected, only the subset of variables for which there are clear soil quality guidelines [32] available were used for the random forest models.

	Chemical	Physical
Measured and used in models	pH, carbon (%), total nitrogen (%), anaerobically mineralizable nitrogen (AMN, mg/kg), Olsen P (mg/kg).	Macroporosity (MP, % v/v), bulk density (BD, t/m^3^).
Measured but not used in models	C:N, NO3-M (mg/kg), NH4-N (mg/kg), Arsenic* (mg/kg), Cadmium* (mg/kg), Chromium* (mg/kg), Copper* (mg/kg), Nickel* (mg/kg), Lead* (mg/kg), Zinc* (mg/kg).	

*There are clear guidelines around the concentration of metals deemed acceptable, but an insufficient number of sites (< 5%) in the dataset had ‘contaminated’ soils; therefore, these variables were not modelled.

### Molecular methods

To ensure soil samples for molecular analysis were processed in a similar manner to those collected and analysed for soil physico-chemical attributes, soil samples including bulk soil, plant roots and other biomass were processed in their entirety. Individual soil cores were manually homogenised and DNA extracted from 25 mg of soil using the PowerSoil-htp/DNeasy PowerSoil-htp DNA extraction kit (Mo Bio Laboratories Inc. or Qiagen, respectively). DNA extractions were performed as per manufacturer’s instructions, except that mechanical lysis was performed by agitating the plates in a Qiagen TissueLyser II (Retch) for 4 min at 30 Hz and plates were incubated at room temperature for 5 min after adding the elution buffer, prior to the final centrifuge. In total, DNA was extracted from 3,030 samples, which were stored at − 20 °C.

Bacterial communities have been shown to respond strongly to changes in their soil environment, especially variables directly related to soil quality [11, 14, 17]. Furthermore, there are well-established molecular methods for determining the composition of bacteria within the soil environment. Bacteria therefore make ideal candidates for the exploration of biological indicators at large scales. The V3-V4 region of the bacterial 16S rRNA gene was amplified from each DNA extract as described previously [52]. Normalised PCR products were barcoded (Nextera XT dual indices, Illumina Inc., USA), pooled, and sequenced on an Illumina MiSeq instrument using V3 chemistry to generate 2x300 bp reads. Multiple sequencing runs were performed, each with ~ 384 samples.

### Bioinformatics and statistical analyses

Sequence data were processed as described previously [52] by using USEARCH v 7.0 [53] to filter sequences, remove chimeras, cluster into operational taxonomic units (OTUs) at 97% sequence similarity, and classify against the Greengenes reference database v13.8 [54]. A very small portion of OTUs (0.02%) were classified as unknown Archaea. While we chose not to remove these OTUs from our dataset, we refer to ‘bacterial communities’ in this manuscript given the pre-dominance of bacterial taxa within our dataset.

All statistical analyses and data visualisations were performed in R v3.6.1 (R Core Team 2016). Prior to all analyses, the OTU table was rarefied to 2000 reads per sample using the ‘rarefy’ function in the ‘vegan’ package [55], to ensure sequencing depth was comparable across all samples. Furthermore, the replicate data for each site (*n* = 5) were averaged to obtain one representative bacterial community per site. This was necessary as the soil physico-chemical data were measured for composite soil collected across the entire transect, rather than for each individual soil sample. The ‘vegan’ package was also used to compute a Bray-Curtis dissimilarity matrix to compare the bacterial communities among all sites. Differences in bacterial community composition between land uses were visualised using non-metric multidimensional scaling and tested using PERMANOVA with 999 permutations.

After rarefying, there were 42,812 OTUs across all the samples. To reduce the number of explanatory variables entering each random forest model, the OTU table was filtered to select OTUs which best represented differences amongst the samples. For this, samples were clustered according to the composition of their bacterial communities using Ward’s minimum variance clustering; this step was performed on three variants of the OTU table, corresponding to the three different datasets that were modelled (Fig. 1). The first OTU table contained sample data from all sites (indigenous, exotic, horticulture and pastoral grassland), the second all managed (AM) sites (exotic, horticulture and pastoral grassland), and the third only from non-pastoral grassland managed (NPG) sites (exotic and horticulture). To interpret and compare clusters, a single cutting level for each dendrogram was picked. To judge which cut off level was the most appropriate, several criteria were assessed: silhouette width, dissimilarity and binary matrix correlation, and species fidelity analyses (see [56] for detailed explanations). Ultimately, the cut off level was based on the optimal level as determined by these parameters, while maintaining an adequate number of samples per cluster for downstream analyses. For the OTU table with all sites, five clusters were created, while for the AM and NPG OTU tables, seven clusters were used. To select representative OTUs for each cluster, the ‘indicspecies’ package was used [57]. Using the ‘indicators’ command, we selected OTUs which were indicators of each data cluster with At (specificity) and Bt (fidelity) scores of > 0.4 for the five ‘all samples’ clusters and > 0.5 for the seven AM and NPG clusters. This gave 648, 688 and 830 OTUs for all the sites, AM sites and NPG sites, respectively. These OTUs were then used as explanatory variables for the random forest analyses.

Ward’s minimum variance clustering was also used to cluster the managed sites based on the soil conditions at each site (Fig. 1). This was performed as per the OTU-based clustering, except with the soil variables in row one of Table 1. For the AM subset, this resulted in five clusters, while for the NPG subset there were four clusters (Fig. S9, Additional file 1). Dunn’s test for multiple comparisons with Bonferroni corrections was used to determine how the soil variables differed among the different clusters and could therefore be used as descriptors for the sites in those clusters. Additionally, a Bayesian PCA was performed using the ‘pcaMethods’ package [58] to obtain PCA scores for each site based on the soil chemistry.

There are many different machine learning approaches that can be used to create predictive models, each with their strengths and weaknesses [59]. Here, we use Random forest analyses [28]. This method has previously been shown to outperform other modelling approaches when used for environmental bacterial datasets [29]. Random forest analyses were performed using the ‘randomForest’ package with default parameters (Liaw and Wiener 2002). Stratified random sampling was used to select 80% of sites from each land use to be used as the training dataset for the models. The random forest models were then validated on the remaining 20% of the sites. Details for the explanatory and response variables used in each model can be found in Fig. 1 and were either qualitative, meaning the algorithm was performed classifications, or quantitative meaning regressions were performed. The ‘varImpPlot’ command was used to obtain the top 15 most important OTUs for each model based on the decrease in mean squared error (% Inc. MSE) when those OTUs are included. While these OTUs should not be considered indicator species, as alone they are not able to predict the soil characteristics, they can be considered important to the model’s overall success.

Linear regression models were used to assess the accuracy of the random forest predictions for quantitative response variables; *R*^2^ and slope values closer to 1 indicate better models. Additionally, predicted soil environmental variables were converted to soil quality scores. These scores were based on guidelines as detailed by Hill and Sparling (2009; see Tables S3–S9, Additional file 1 for more details). The predicted soil quality scores were compared to the true scores for each site, to determine the extent to which the random forest models can be used to indicate the quality of managed soils.

## Supplementary information

**Additional file 1.** Additional information.

## Data Availability

The dataset supporting the conclusions of this article is available in the NCBI Sequence Read Archive repository under accession number PRJNA578562. This study also uses the previously published sequence data in PRJNA323375.
