# Supplementary material for: Using soil bacterial communities to predict physico-chemical variables and soil quality
Source: Microbiome. 2020 Jun 2;8:79. doi: 10.1186/s40168-020-00858-1 (PMC7268603; doi:10.1186/s40168-020-00858-1)
Supplement: Supplementary file 2 — Additional file 1. Additional information. [file 40168_2020_858_MOESM1_ESM.docx]

**ADDITIONAL INFORMATION**

**
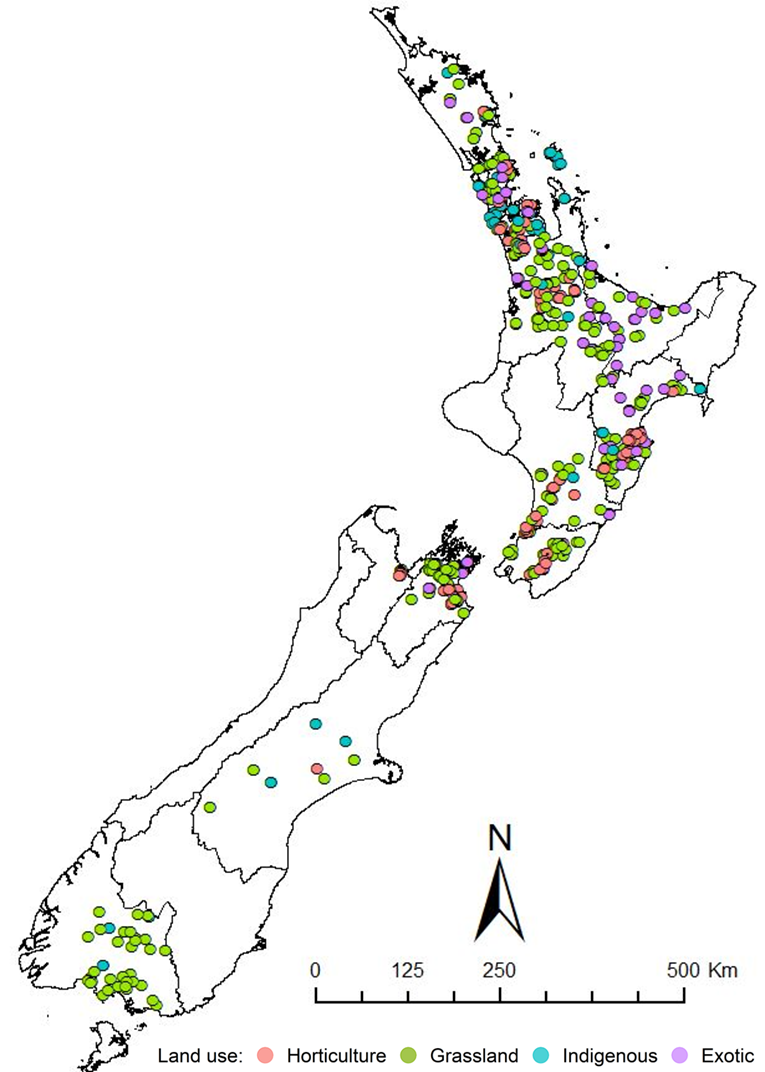
**

Figure S1: The location of the sites that were sampled. Sites were classified as belonging to one of four land uses: indigenous forest, exotic plantation forest, horticulture or pastoral grassland.


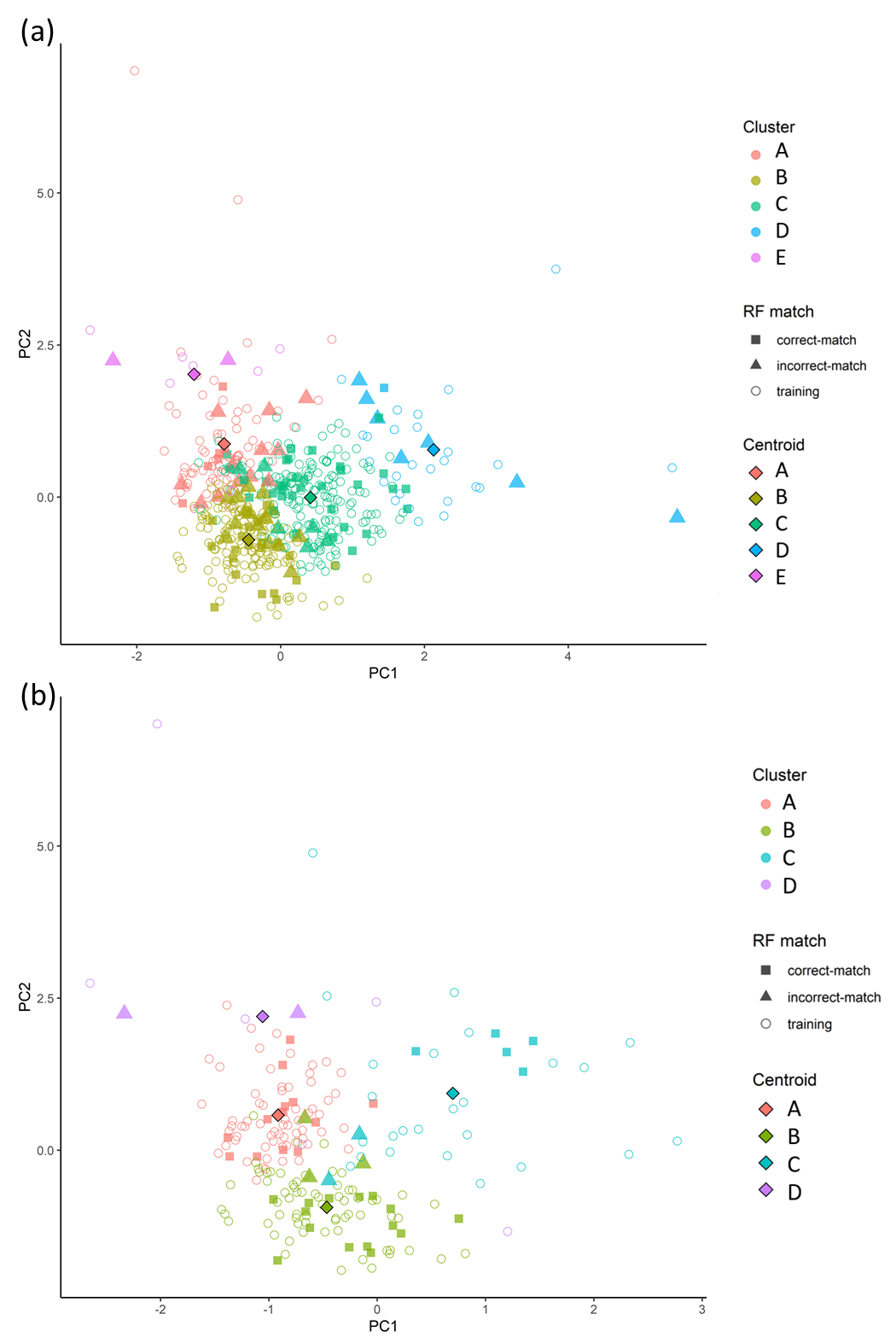


Figure S2: Underlying differences in soil physicochemical variables at (a) all the managed sites or (b) all the non-pastoral grassland sites. Sites are coloured according to the chemistry cluster to which they belonged; shapes indicate whether a site was assigned the correct cluster.


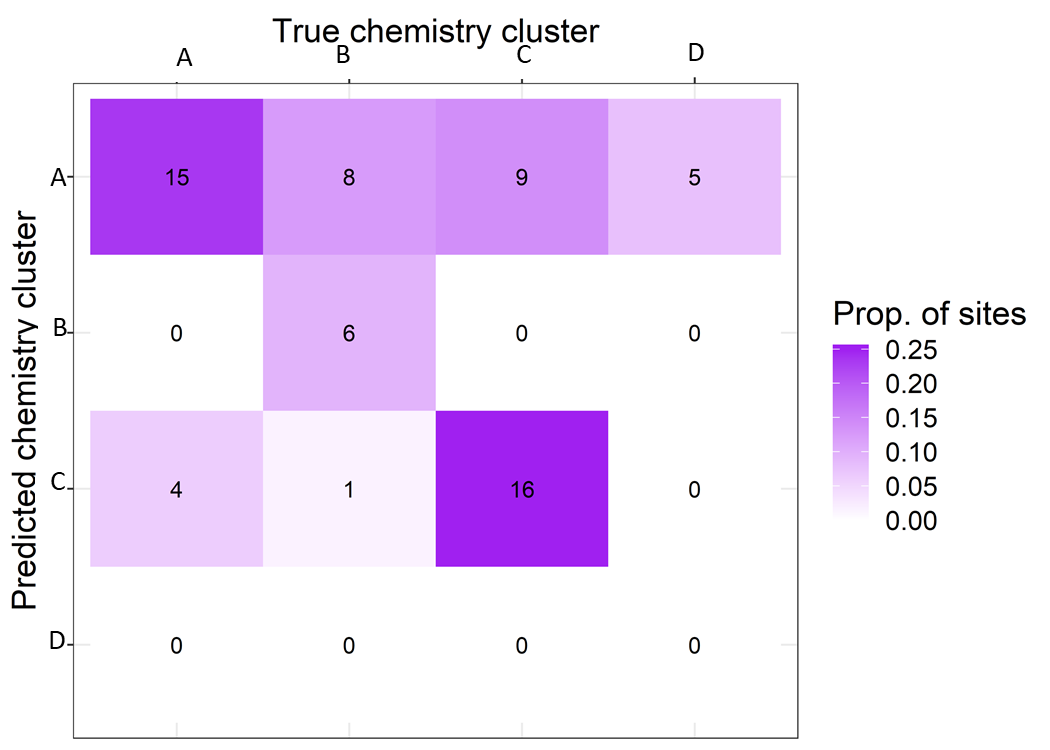


Figure S3: The number of correct and incorrect predictions of soil chemistry cluster for the pastoral grassland sites, based on a random forest classification model.


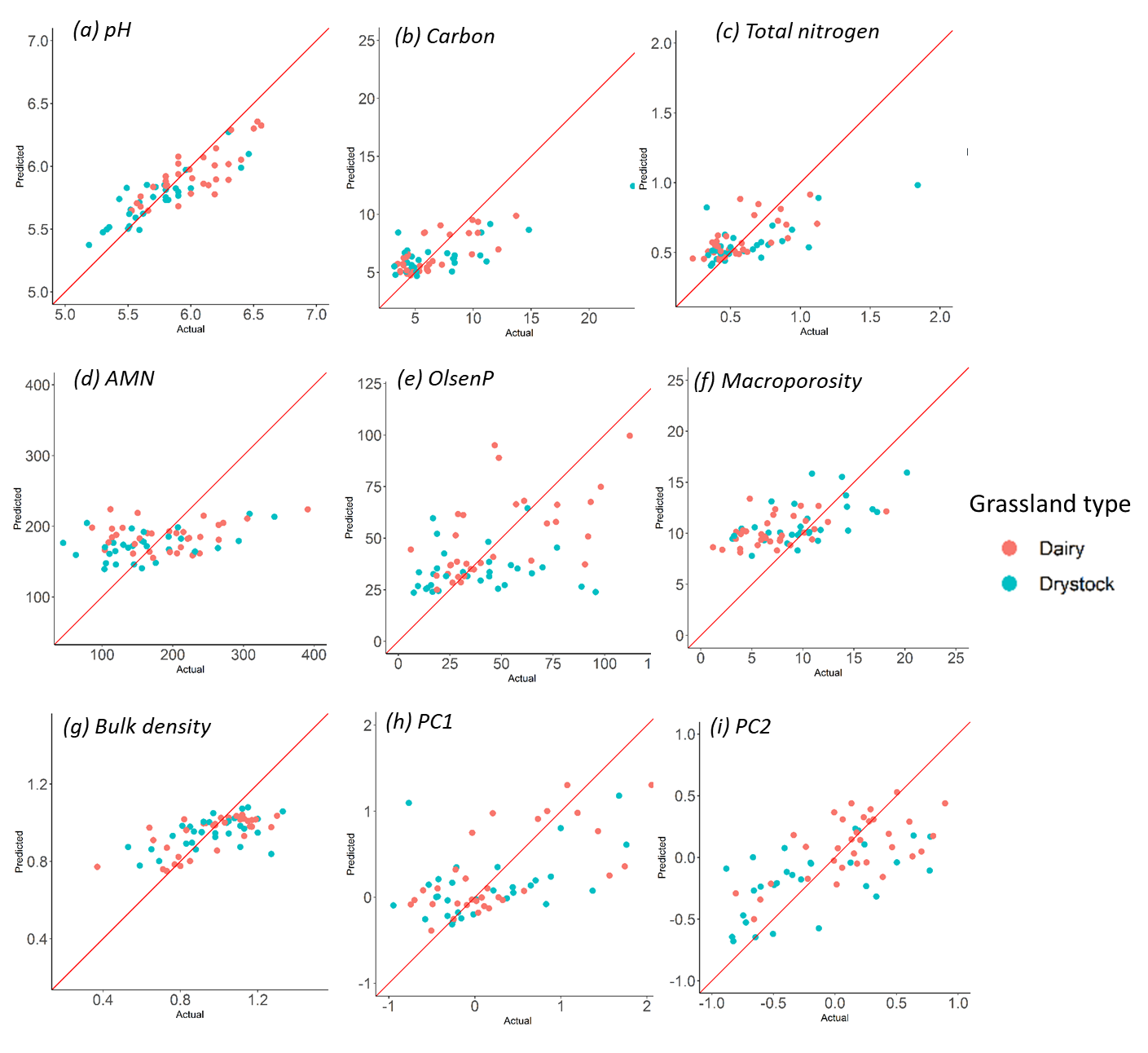


Figure S4: Predicted (a-g) soil variable values or (h-i) principal component analyses axes scores versus actual values for pastoral grassland sites. Red lines indicate where points should fall for a perfect prediction.


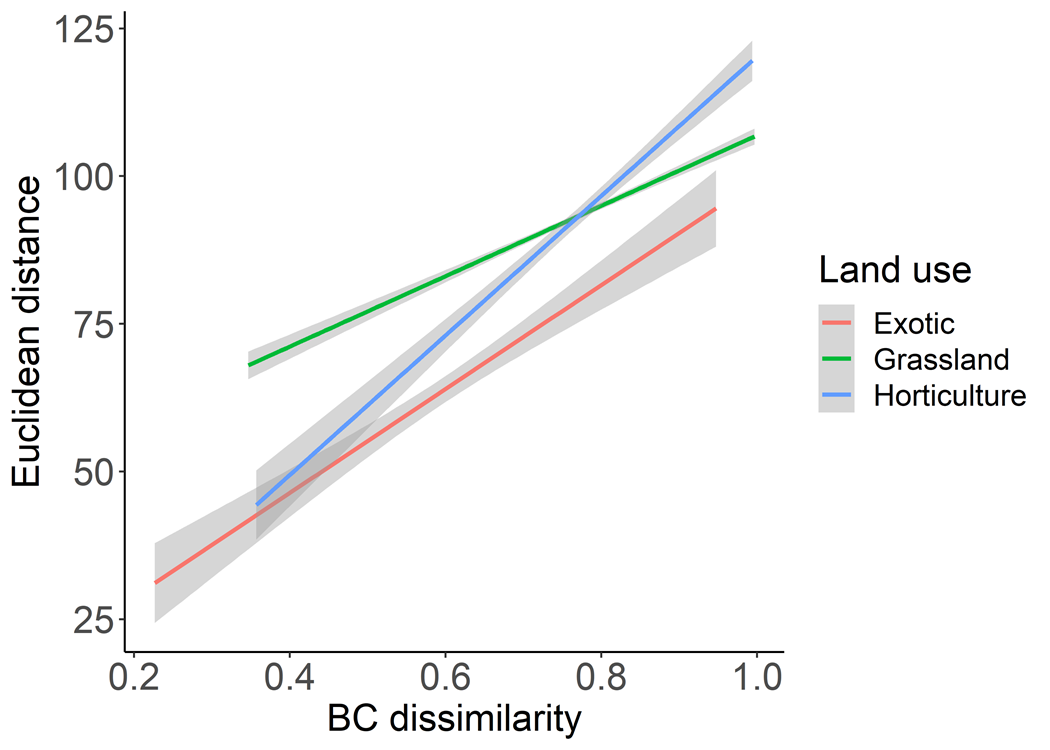


Figure S5: The correlation between Bray-Curtis dissimilarity of soil bacterial community composition with dissimilarity in the soil variables (Euclidean distance) for the different land uses .


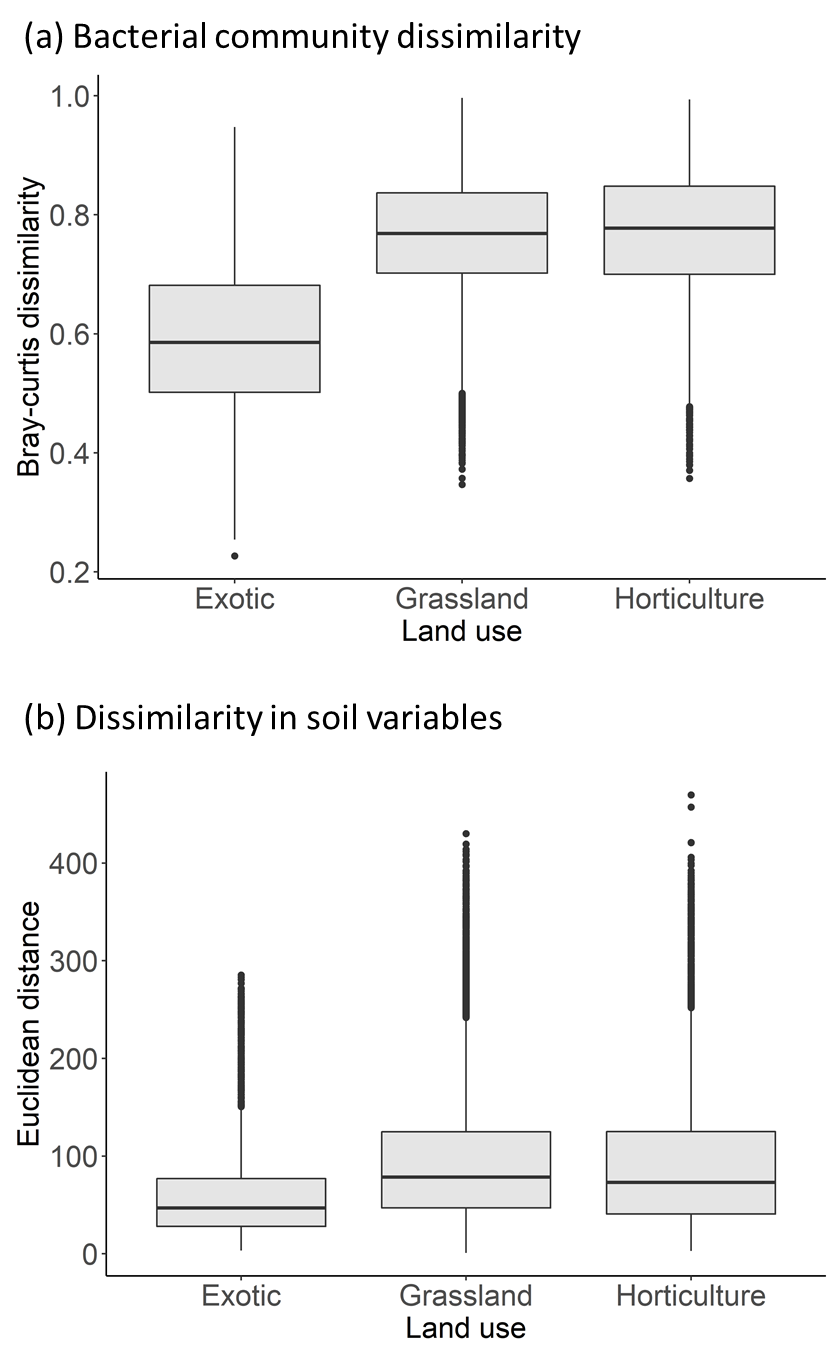


Figure S6: The pairwise dissimilarity in (a) bacterial community composition and (b) soil environment for samples from the same land use. Boxes represent the interquartile range (IQR, 25-75% of the data). Median values are indicated by the bar within each box and whiskers show the values within 1.5 times the IQR; all other values are outliers and are shown as points.


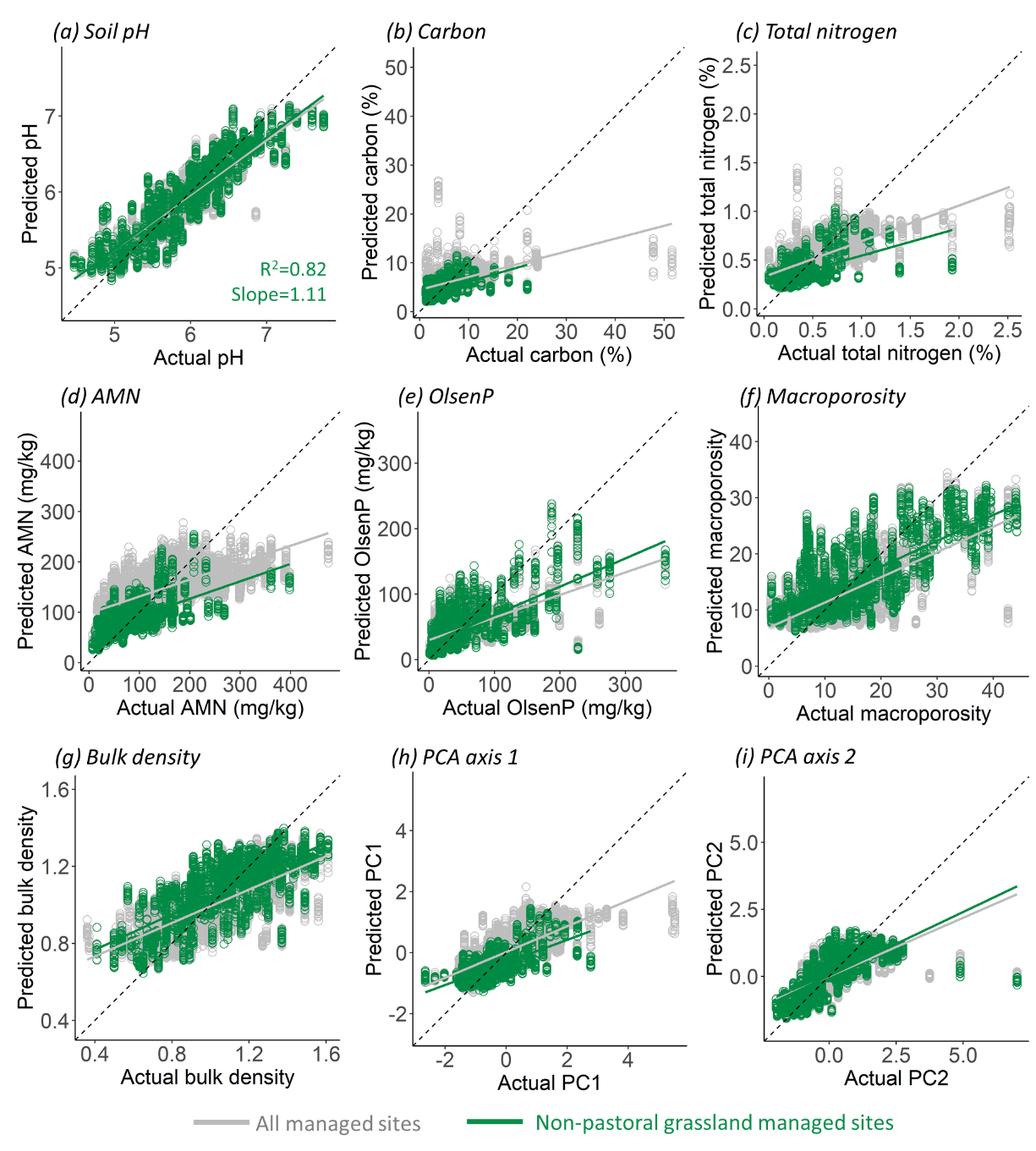


Figure S7: Predicted (a-g) soil variable values or (h-i) principal component analyses axes scores versus actual values, based on 100 iterations of the random forest regression analyses, using a different random subset of samples each time. Models were based on either (in grey) all sites belonging to a managed land use type (horticulture, exotic or pastoral grassland) or (in green) sites belonging to non-pastoral grassland managed land uses (NPG). Dashed black lines indicate where points should fall for a perfect prediction.


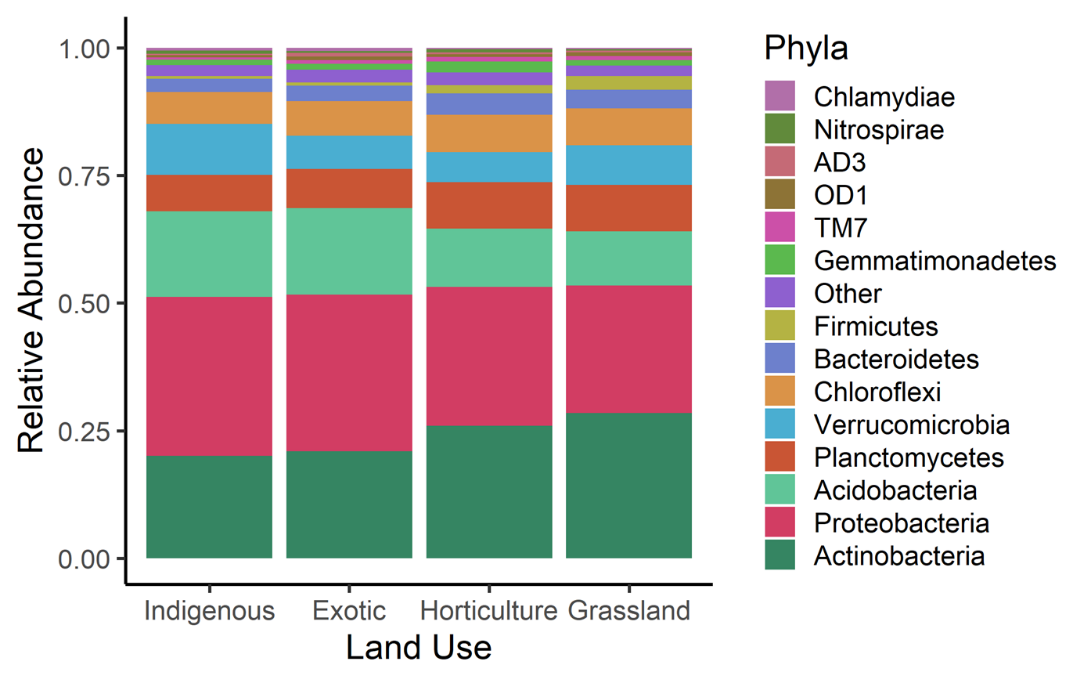


Figure S8: The relative abundances of the most dominant phyla present at the sites, grouped by land use.

Table S1: The taxonomic classifications of the OTUs which were the most important for each random forest model using the “AM” dataset. Only the top 15 most important OTUs are shown for each model. Numbers indicate the order of importance, with 1 being the most important.

|  |  | Random forest model | | | | | | | | |
| --- | --- | --- | --- | --- | --- | --- | --- | --- | --- | --- |
| OTU | **Taxonomy** | **PC1** | **PC2** | **pH** | **C** | **TN** | **AMN** | **OlsenP** | **MP** | **BD** |
| OTU_100 | p__Acidobacteria c__Acidobacteriia o__Acidobacteriales f__Acidobacteriaceae | 12 |  |  |  | 11 |  | 7 |  |  |
| OTU_1019 | p__Acidobacteria c__Solibacteres o__Solibacterales |  | 12 |  |  |  |  |  |  |  |
| OTU_1041 | p__Bacteroidetes c__Sphingobacteriia o__Sphingobacteriales f__Sphingobacteriaceae |  |  |  |  |  |  |  | 2 |  |
| OTU_106 | p__Chloroflexi c__Ktedonobacteria o__Thermogemmatisporales f__Thermogemmatisporaceae | 15 |  |  |  |  |  |  |  |  |
| OTU_1063 | p__Acidobacteria c__[Chloracidobacteria] o__RB41 |  |  |  | 10 |  | 6 |  |  |  |
| OTU_1066 | p__Verrucomicrobia c__[Pedosphaerae] o__[Pedosphaerales] f__auto67_4W |  | 13 |  |  |  |  |  |  |  |
| OTU_10666 | p__Acidobacteria c__Acidobacteria-6 o__iii1-15 |  |  | 3 |  |  |  |  |  |  |
| OTU_1134 | p__Acidobacteria c__Acidobacteriia o__Acidobacteriales f__Koribacteraceae |  |  |  |  |  |  | 3 |  |  |
| OTU_114 | p__Actinobacteria c__Actinobacteria o__Actinomycetales | 13 |  |  | 5 | 15 |  |  |  | 11 |
| OTU_117 | p__Proteobacteria c__Gammaproteobacteria o__Xanthomonadales f__Sinobacteraceae |  |  |  |  |  |  |  | 1 |  |
| OTU_1172 | p__Acidobacteria c__Acidobacteria-6 o__iii1-15 |  |  | 4 |  |  |  |  |  |  |
| OTU_1189 | p__Actinobacteria c__Actinobacteria o__Actinomycetales |  |  | 7 |  |  |  |  |  |  |
| OTU_1213 | p__Verrucomicrobia c__[Pedosphaerae] o__[Pedosphaerales] f__[Pedosphaeraceae] g__Pedosphaera |  | 9 |  |  |  |  |  |  |  |
| OTU_12651 | p__Verrucomicrobia c__[Spartobacteria] o__[Chthoniobacterales] f__[Chthoniobacteraceae] g__DA101 | 1 |  |  | 1 | 1 | 5 |  |  | 1 |
| OTU_1288 | p__Planctomycetes c__Planctomycetia o__Pirellulales f__Pirellulaceae |  |  | 9 |  |  |  |  |  |  |
| OTU_1293 | p__Proteobacteria c__Betaproteobacteria o__SC-I-84 |  |  |  |  | 14 |  | 8 |  |  |
| OTU_136 | p__Bacteroidetes c__Cytophagia o__Cytophagales f__Cytophagaceae |  |  | 14 |  |  |  |  |  |  |
| OTU_140 | p__Actinobacteria c__Actinobacteria o__Actinomycetales f__Mycobacteriaceae g__Mycobacterium |  |  |  |  |  |  |  | 7 |  |
| OTU_1430 | p__Verrucomicrobia c__[Spartobacteria] o__[Chthoniobacterales] f__[Chthoniobacteraceae] g__DA101 |  |  |  |  |  |  | 2 |  |  |
| OTU_14923 | p__Actinobacteria c__Acidimicrobiia o__Acidimicrobiales |  |  |  |  |  |  |  |  | 14 |
| OTU_15907 | p__Proteobacteria c__Alphaproteobacteria o__Rhizobiales f__Methylocystaceae |  | 10 |  |  |  |  |  |  |  |
| OTU_164 | p__Proteobacteria c__Alphaproteobacteria o__Rhodospirillales f__Rhodospirillaceae | 3 |  |  | 4 | 2 | 3 | 4 |  |  |
| OTU_1732 | p__Gemmatimonadetes c__Gemmatimonadetes o__N1423WL |  |  |  |  |  | 12 |  |  |  |
| OTU_177 | p__Proteobacteria c__Betaproteobacteria o__Burkholderiales f__Burkholderiaceae g__Burkholderia |  |  |  |  |  | 9 |  |  |  |
| OTU_1880 | p__Chloroflexi c__C0119 |  |  |  |  |  |  | 13 |  |  |
| OTU_191 | p__Actinobacteria c__Actinobacteria o__Actinomycetales f__Mycobacteriaceae g__Mycobacterium |  |  |  |  |  |  |  | 5 |  |
| OTU_20374 | p__Actinobacteria c__Actinobacteria o__Actinomycetales |  |  |  |  |  |  |  | 14 |  |
| OTU_2041 | p__Nitrospirae c__Nitrospira o__Nitrospirales f__FW g__4-29 |  | 8 |  |  |  |  |  |  |  |
| OTU_21069 | p__Actinobacteria c__Acidimicrobiia o__Acidimicrobiales |  | 14 |  |  |  |  |  |  |  |
| OTU_2143 | p__Bacteroidetes c__[Saprospirae] o__[Saprospirales] f__Chitinophagaceae g__Flavisolibacter |  |  |  |  |  | 7 |  |  |  |
| OTU_225 | p__Acidobacteria c__DA052 o__Ellin6513 |  |  |  |  |  | 11 |  |  |  |
| OTU_230 | p__Planctomycetes c__Phycisphaerae o__WD2101 |  | 7 |  |  |  |  |  |  |  |
| OTU_231 | p__Acidobacteria c__DA052 o__Ellin6513 | 11 |  |  | 13 |  |  |  | 4 |  |
| OTU_24 | p__Verrucomicrobia c__[Spartobacteria] o__[Chthoniobacterales] f__[Chthoniobacteraceae] g__Candidatus_Xiphinematobacter |  | 2 |  |  |  |  |  | 8 |  |
| OTU_2448 | p__Verrucomicrobia c__[Spartobacteria] o__[Chthoniobacterales] f__[Chthoniobacteraceae] g__DA101 | 14 |  |  | 6 | 6 |  |  |  | 12 |
| OTU_25 | p__Proteobacteria c__Alphaproteobacteria o__Rhodospirillales f__Rhodospirillaceae |  |  |  |  |  |  | 12 |  |  |
| OTU_2503 | p__Proteobacteria c__Alphaproteobacteria |  |  |  |  |  |  | 14 |  |  |
| OTU_2507 | p__Proteobacteria c__Deltaproteobacteria o__NB1-j f__NB1-i |  |  |  | 9 |  |  |  |  |  |
| OTU_2693 | p__Chloroflexi c__S085 |  |  |  |  |  |  | 10 |  |  |
| OTU_2754 | p__Gemmatimonadetes c__Gemmatimonadetes o__N1423WL | 2 |  |  | 2 | 8 | 1 |  |  | 2 |
| OTU_293 | p__Actinobacteria c__Acidimicrobiia o__Acidimicrobiales |  |  |  | 15 |  |  |  |  |  |
| OTU_300 | p__Planctomycetes c__Phycisphaerae o__WD2101 |  |  |  |  |  |  |  |  | 15 |
| OTU_301 | p__Proteobacteria c__Alphaproteobacteria o__Rhodospirillales f__Rhodospirillaceae | 10 |  |  | 11 |  | 2 |  |  |  |
| OTU_3157 | p__Acidobacteria c__Solibacteres o__Solibacterales |  |  |  |  | 12 |  |  |  |  |
| OTU_342 | p__Chloroflexi c__Thermomicrobia o__JG30-KF-CM45 |  |  |  | 12 |  |  |  |  |  |
| OTU_34454 | p__Planctomycetes c__Phycisphaerae o__WD2101 | 5 |  |  | 8 | 7 |  |  |  | 4 |
| OTU_372 | p__Planctomycetes c__Phycisphaerae o__WD2101 |  |  |  |  |  |  |  | 9 |  |
| OTU_404 | p__Proteobacteria c__Alphaproteobacteria o__Rhizobiales f__Hyphomicrobiaceae g__Pedomicrobium |  |  | 11 |  |  |  |  |  |  |
| OTU_45245 | p__Bacteroidetes c__[Saprospirae] o__[Saprospirales] f__Chitinophagaceae |  |  |  |  |  |  |  | 10 |  |
| OTU_458 | p__Acidobacteria c__[Chloracidobacteria] o__RB41 f__Ellin6075 |  |  |  |  |  |  |  |  | 13 |
| OTU_47493 | p__Acidobacteria c__Acidobacteriia o__Acidobacteriales f__Koribacteraceae |  |  |  |  |  |  |  |  | 7 |
| OTU_4831 | p__Actinobacteria c__Thermoleophilia o__Solirubrobacterales f__Conexibacteraceae g__Conexibacter |  |  |  |  |  |  |  | 12 |  |
| OTU_499 | p__Actinobacteria c__Thermoleophilia o__Gaiellales |  |  |  |  |  |  |  | 11 |  |
| OTU_5275 | p__Gemmatimonadetes c__Gemmatimonadetes o__Ellin5290 |  |  |  |  |  |  |  |  | 6 |
| OTU_5310 | p__Nitrospirae c__Nitrospira o__Nitrospirales f__Nitrospiraceae g__Nitrospira |  |  |  |  |  |  | 1 |  |  |
| OTU_532 | p__Proteobacteria c__Alphaproteobacteria o__Rhodospirillales f__Acetobacteraceae |  |  |  |  | 9 |  |  |  |  |
| OTU_5436 | p__Proteobacteria c__Betaproteobacteria o__Burkholderiales f__Comamonadaceae g__Ramlibacter |  |  |  |  |  |  |  | 6 |  |
| OTU_551 | p__Proteobacteria c__Alphaproteobacteria o__Rhodospirillales f__Rhodospirillaceae |  | 11 |  |  |  |  |  |  |  |
| OTU_56 | p__Actinobacteria c__Acidimicrobiia o__Acidimicrobiales |  |  | 12 |  |  |  |  |  |  |
| OTU_565 | p__Verrucomicrobia c__[Spartobacteria] o__[Chthoniobacterales] f__[Chthoniobacteraceae] |  | 15 |  |  |  |  |  |  |  |
| OTU_597 | p__Proteobacteria c__Gammaproteobacteria o__Xanthomonadales f__Xanthomonadaceae | 9 |  |  |  |  | 8 |  |  | 10 |
| OTU_6009 | p__Acidobacteria c__Acidobacteria-6 o__iii1-15 |  |  |  |  |  | 14 | 5 |  |  |
| OTU_6056 | p__Proteobacteria c__Alphaproteobacteria o__Caulobacterales f__Caulobacteraceae |  |  |  |  | 10 | 10 |  |  |  |
| OTU_6093 | p__Chloroflexi c__Ellin6529 | 8 |  |  | 7 | 13 | 4 |  | 3 | 9 |
| OTU_63365 | p__Acidobacteria c__Acidobacteria-6 o__iii1-15 |  |  | 5 |  |  |  |  |  |  |
| OTU_64 | p__Acidobacteria c__DA052 o__Ellin6513 |  |  |  |  |  |  |  | 13 |  |
| OTU_65 | p__Verrucomicrobia c__[Spartobacteria] o__[Chthoniobacterales] f__[Chthoniobacteraceae] g__Candidatus_Xiphinematobacter |  | 5 |  |  |  |  |  |  |  |
| OTU_6567 | p__Acidobacteria c__Acidobacteria-6 o__iii1-15 |  |  | 15 |  |  |  |  |  |  |
| OTU_68 | p__Acidobacteria c__Acidobacteriia o__Acidobacteriales f__Acidobacteriaceae |  |  | 1 |  |  |  | 11 |  |  |
| OTU_6865 | p__Verrucomicrobia c__[Spartobacteria] o__[Chthoniobacterales] f__[Chthoniobacteraceae] g__DA101 | 6 |  |  |  | 5 | 13 |  |  | 3 |
| OTU_699 | p__Proteobacteria c__Alphaproteobacteria o__Rhodospirillales f__Rhodospirillaceae |  |  |  |  |  |  | 9 |  |  |
| OTU_715 | p__Planctomycetes c__Planctomycetia o__Pirellulales f__Pirellulaceae |  |  | 13 |  |  |  |  |  |  |
| OTU_74 | p__Actinobacteria c__Actinobacteria o__Actinomycetales |  |  | 10 |  |  |  | 6 |  |  |
| OTU_746 | p__Acidobacteria c__Acidobacteria-6 o__iii1-15 |  |  | 6 |  |  |  |  |  |  |
| OTU_7821 | p__Proteobacteria c__Betaproteobacteria o__SC-I-84 | 4 |  |  | 3 | 3 |  |  |  | 5 |
| OTU_807 | p__Acidobacteria c__Acidobacteriia o__Acidobacteriales f__Acidobacteriaceae |  | 4 |  |  |  |  |  |  |  |
| OTU_8584 | p__Acidobacteria c__Acidobacteria-6 o__iii1-15 |  |  | 8 |  |  |  |  |  |  |
| OTU_877 | p__Proteobacteria c__Gammaproteobacteria o__Xanthomonadales f__Sinobacteraceae |  | 6 |  |  |  |  |  |  |  |
| OTU_9 | p__Proteobacteria c__Alphaproteobacteria o__Rhizobiales f__Methylocystaceae |  | 1 | 2 |  |  |  |  |  |  |
| OTU_90 | p__Proteobacteria c__Alphaproteobacteria o__Caulobacterales f__Caulobacteraceae |  |  |  |  |  |  |  | 15 |  |
| OTU_917 | p__Chloroflexi c__Anaerolineae o__envOPS12 |  |  |  |  |  |  | 15 |  |  |
| OTU_980 | p__Verrucomicrobia c__[Spartobacteria] o__[Chthoniobacterales] f__[Chthoniobacteraceae] g__DA101 | 7 |  |  | 14 | 4 | 15 |  |  | 8 |
| OTU_99 | p__Proteobacteria c__Gammaproteobacteria o__Xanthomonadales f__Sinobacteraceae |  | 3 |  |  |  |  |  |  |  |

Table S2: The taxonomic classifications of the OTUs which were the most important for each random forest model using the “NPG” dataset. Only the top 15 most important OTUs are shown for each model. Numbers indicate the order of importance, with 1 being the most important.

|  |  | Random forest model | | | | | | | | |
| --- | --- | --- | --- | --- | --- | --- | --- | --- | --- | --- |
| OTU | **Taxonomy** | **PC1** | **PC2** | **pH** | **C** | **TN** | **AMN** | **OlsenP** | **MP** | **BD** |
| OTU_100 | p__Acidobacteria c__Acidobacteriia o__Acidobacteriales f__Acidobacteriaceae | 12 |  |  |  | 11 |  | 7 |  |  |
| OTU_1019 | p__Acidobacteria c__Solibacteres o__Solibacterales |  | 12 |  |  |  |  |  |  |  |
| OTU_1041 | p__Bacteroidetes c__Sphingobacteriia o__Sphingobacteriales f__Sphingobacteriaceae |  |  |  |  |  |  |  | 2 |  |
| OTU_106 | p__Chloroflexi c__Ktedonobacteria o__Thermogemmatisporales f__Thermogemmatisporaceae | 15 |  |  |  |  |  |  |  |  |
| OTU_1063 | p__Acidobacteria c__[Chloracidobacteria] o__RB41 |  |  |  | 10 |  | 6 |  |  |  |
| OTU_1066 | p__Verrucomicrobia c__[Pedosphaerae] o__[Pedosphaerales] f__auto67_4W |  | 13 |  |  |  |  |  |  |  |
| OTU_10666 | p__Acidobacteria c__Acidobacteria-6 o__iii1-15 |  |  | 3 |  |  |  |  |  |  |
| OTU_1134 | p__Acidobacteria c__Acidobacteriia o__Acidobacteriales f__Koribacteraceae |  |  |  |  |  |  | 3 |  |  |
| OTU_114 | p__Actinobacteria c__Actinobacteria o__Actinomycetales | 13 |  |  | 5 | 15 |  |  |  | 11 |
| OTU_117 | p__Proteobacteria c__Gammaproteobacteria o__Xanthomonadales f__Sinobacteraceae |  |  |  |  |  |  |  | 1 |  |
| OTU_1172 | p__Acidobacteria c__Acidobacteria-6 o__iii1-15 |  |  | 4 |  |  |  |  |  |  |
| OTU_1189 | p__Actinobacteria c__Actinobacteria o__Actinomycetales |  |  | 7 |  |  |  |  |  |  |
| OTU_1213 | p__Verrucomicrobia c__[Pedosphaerae] o__[Pedosphaerales] f__[Pedosphaeraceae] g__Pedosphaera |  | 9 |  |  |  |  |  |  |  |
| OTU_12651 | p__Verrucomicrobia c__[Spartobacteria] o__[Chthoniobacterales] f__[Chthoniobacteraceae] g__DA101 | 1 |  |  | 1 | 1 | 5 |  |  | 1 |
| OTU_1288 | p__Planctomycetes c__Planctomycetia o__Pirellulales f__Pirellulaceae |  |  | 9 |  |  |  |  |  |  |
| OTU_1293 | p__Proteobacteria c__Betaproteobacteria o__SC-I-84 |  |  |  |  | 14 |  | 8 |  |  |
| OTU_136 | p__Bacteroidetes c__Cytophagia o__Cytophagales f__Cytophagaceae |  |  | 14 |  |  |  |  |  |  |
| OTU_140 | p__Actinobacteria c__Actinobacteria o__Actinomycetales f__Mycobacteriaceae g__Mycobacterium |  |  |  |  |  |  |  | 7 |  |
| OTU_1430 | p__Verrucomicrobia c__[Spartobacteria] o__[Chthoniobacterales] f__[Chthoniobacteraceae] g__DA101 |  |  |  |  |  |  | 2 |  |  |
| OTU_14923 | p__Actinobacteria c__Acidimicrobiia o__Acidimicrobiales |  |  |  |  |  |  |  |  | 14 |
| OTU_15907 | p__Proteobacteria c__Alphaproteobacteria o__Rhizobiales f__Methylocystaceae |  | 10 |  |  |  |  |  |  |  |
| OTU_164 | p__Proteobacteria c__Alphaproteobacteria o__Rhodospirillales f__Rhodospirillaceae | 3 |  |  | 4 | 2 | 3 | 4 |  |  |
| OTU_1732 | p__Gemmatimonadetes c__Gemmatimonadetes o__N1423WL |  |  |  |  |  | 12 |  |  |  |
| OTU_177 | p__Proteobacteria c__Betaproteobacteria o__Burkholderiales f__Burkholderiaceae g__Burkholderia |  |  |  |  |  | 9 |  |  |  |
| OTU_1880 | p__Chloroflexi c__C0119 |  |  |  |  |  |  | 13 |  |  |
| OTU_191 | p__Actinobacteria c__Actinobacteria o__Actinomycetales f__Mycobacteriaceae g__Mycobacterium |  |  |  |  |  |  |  | 5 |  |
| OTU_20374 | p__Actinobacteria c__Actinobacteria o__Actinomycetales |  |  |  |  |  |  |  | 14 |  |
| OTU_2041 | p__Nitrospirae c__Nitrospira o__Nitrospirales f__FW g__4-29 |  | 8 |  |  |  |  |  |  |  |
| OTU_21069 | p__Actinobacteria c__Acidimicrobiia o__Acidimicrobiales |  | 14 |  |  |  |  |  |  |  |
| OTU_2143 | p__Bacteroidetes c__[Saprospirae] o__[Saprospirales] f__Chitinophagaceae g__Flavisolibacter |  |  |  |  |  | 7 |  |  |  |
| OTU_225 | p__Acidobacteria c__DA052 o__Ellin6513 |  |  |  |  |  | 11 |  |  |  |
| OTU_230 | p__Planctomycetes c__Phycisphaerae o__WD2101 |  | 7 |  |  |  |  |  |  |  |
| OTU_231 | p__Acidobacteria c__DA052 o__Ellin6513 | 11 |  |  | 13 |  |  |  | 4 |  |
| OTU_24 | p__Verrucomicrobia c__[Spartobacteria] o__[Chthoniobacterales] f__[Chthoniobacteraceae] g__Candidatus_Xiphinematobacter |  | 2 |  |  |  |  |  | 8 |  |
| OTU_2448 | p__Verrucomicrobia c__[Spartobacteria] o__[Chthoniobacterales] f__[Chthoniobacteraceae] g__DA101 | 14 |  |  | 6 | 6 |  |  |  | 12 |
| OTU_25 | p__Proteobacteria c__Alphaproteobacteria o__Rhodospirillales f__Rhodospirillaceae |  |  |  |  |  |  | 12 |  |  |
| OTU_2503 | p__Proteobacteria c__Alphaproteobacteria |  |  |  |  |  |  | 14 |  |  |
| OTU_2507 | p__Proteobacteria c__Deltaproteobacteria o__NB1-j f__NB1-i |  |  |  | 9 |  |  |  |  |  |
| OTU_2693 | p__Chloroflexi c__S085 |  |  |  |  |  |  | 10 |  |  |
| OTU_2754 | p__Gemmatimonadetes c__Gemmatimonadetes o__N1423WL | 2 |  |  | 2 | 8 | 1 |  |  | 2 |
| OTU_293 | p__Actinobacteria c__Acidimicrobiia o__Acidimicrobiales |  |  |  | 15 |  |  |  |  |  |
| OTU_300 | p__Planctomycetes c__Phycisphaerae o__WD2101 |  |  |  |  |  |  |  |  | 15 |
| OTU_301 | p__Proteobacteria c__Alphaproteobacteria o__Rhodospirillales f__Rhodospirillaceae | 10 |  |  | 11 |  | 2 |  |  |  |
| OTU_3157 | p__Acidobacteria c__Solibacteres o__Solibacterales |  |  |  |  | 12 |  |  |  |  |
| OTU_342 | p__Chloroflexi c__Thermomicrobia o__JG30-KF-CM45 |  |  |  | 12 |  |  |  |  |  |
| OTU_34454 | p__Planctomycetes c__Phycisphaerae o__WD2101 | 5 |  |  | 8 | 7 |  |  |  | 4 |
| OTU_372 | p__Planctomycetes c__Phycisphaerae o__WD2101 |  |  |  |  |  |  |  | 9 |  |
| OTU_404 | p__Proteobacteria c__Alphaproteobacteria o__Rhizobiales f__Hyphomicrobiaceae g__Pedomicrobium |  |  | 11 |  |  |  |  |  |  |
| OTU_45245 | p__Bacteroidetes c__[Saprospirae] o__[Saprospirales] f__Chitinophagaceae |  |  |  |  |  |  |  | 10 |  |
| OTU_458 | p__Acidobacteria c__[Chloracidobacteria] o__RB41 f__Ellin6075 |  |  |  |  |  |  |  |  | 13 |
| OTU_47493 | p__Acidobacteria c__Acidobacteriia o__Acidobacteriales f__Koribacteraceae |  |  |  |  |  |  |  |  | 7 |
| OTU_4831 | p__Actinobacteria c__Thermoleophilia o__Solirubrobacterales f__Conexibacteraceae g__Conexibacter |  |  |  |  |  |  |  | 12 |  |
| OTU_499 | p__Actinobacteria c__Thermoleophilia o__Gaiellales |  |  |  |  |  |  |  | 11 |  |
| OTU_5275 | p__Gemmatimonadetes c__Gemmatimonadetes o__Ellin5290 |  |  |  |  |  |  |  |  | 6 |
| OTU_5310 | p__Nitrospirae c__Nitrospira o__Nitrospirales f__Nitrospiraceae g__Nitrospira |  |  |  |  |  |  | 1 |  |  |
| OTU_532 | p__Proteobacteria c__Alphaproteobacteria o__Rhodospirillales f__Acetobacteraceae |  |  |  |  | 9 |  |  |  |  |
| OTU_5436 | p__Proteobacteria c__Betaproteobacteria o__Burkholderiales f__Comamonadaceae g__Ramlibacter |  |  |  |  |  |  |  | 6 |  |
| OTU_551 | p__Proteobacteria c__Alphaproteobacteria o__Rhodospirillales f__Rhodospirillaceae |  | 11 |  |  |  |  |  |  |  |
| OTU_56 | p__Actinobacteria c__Acidimicrobiia o__Acidimicrobiales |  |  | 12 |  |  |  |  |  |  |
| OTU_565 | p__Verrucomicrobia c__[Spartobacteria] o__[Chthoniobacterales] f__[Chthoniobacteraceae] |  | 15 |  |  |  |  |  |  |  |
| OTU_597 | p__Proteobacteria c__Gammaproteobacteria o__Xanthomonadales f__Xanthomonadaceae | 9 |  |  |  |  | 8 |  |  | 10 |
| OTU_6009 | p__Acidobacteria c__Acidobacteria-6 o__iii1-15 |  |  |  |  |  | 14 | 5 |  |  |
| OTU_6056 | p__Proteobacteria c__Alphaproteobacteria o__Caulobacterales f__Caulobacteraceae |  |  |  |  | 10 | 10 |  |  |  |
| OTU_6093 | p__Chloroflexi c__Ellin6529 | 8 |  |  | 7 | 13 | 4 |  | 3 | 9 |
| OTU_63365 | p__Acidobacteria c__Acidobacteria-6 o__iii1-15 |  |  | 5 |  |  |  |  |  |  |
| OTU_64 | p__Acidobacteria c__DA052 o__Ellin6513 |  |  |  |  |  |  |  | 13 |  |
| OTU_65 | p__Verrucomicrobia c__[Spartobacteria] o__[Chthoniobacterales] f__[Chthoniobacteraceae] g__Candidatus_Xiphinematobacter |  | 5 |  |  |  |  |  |  |  |
| OTU_6567 | p__Acidobacteria c__Acidobacteria-6 o__iii1-15 |  |  | 15 |  |  |  |  |  |  |
| OTU_68 | p__Acidobacteria c__Acidobacteriia o__Acidobacteriales f__Acidobacteriaceae |  |  | 1 |  |  |  | 11 |  |  |
| OTU_6865 | p__Verrucomicrobia c__[Spartobacteria] o__[Chthoniobacterales] f__[Chthoniobacteraceae] g__DA101 | 6 |  |  |  | 5 | 13 |  |  | 3 |
| OTU_699 | p__Proteobacteria c__Alphaproteobacteria o__Rhodospirillales f__Rhodospirillaceae |  |  |  |  |  |  | 9 |  |  |
| OTU_715 | p__Planctomycetes c__Planctomycetia o__Pirellulales f__Pirellulaceae |  |  | 13 |  |  |  |  |  |  |
| OTU_74 | p__Actinobacteria c__Actinobacteria o__Actinomycetales |  |  | 10 |  |  |  | 6 |  |  |
| OTU_746 | p__Acidobacteria c__Acidobacteria-6 o__iii1-15 |  |  | 6 |  |  |  |  |  |  |
| OTU_7821 | p__Proteobacteria c__Betaproteobacteria o__SC-I-84 | 4 |  |  | 3 | 3 |  |  |  | 5 |
| OTU_807 | p__Acidobacteria c__Acidobacteriia o__Acidobacteriales f__Acidobacteriaceae |  | 4 |  |  |  |  |  |  |  |
| OTU_8584 | p__Acidobacteria c__Acidobacteria-6 o__iii1-15 |  |  | 8 |  |  |  |  |  |  |
| OTU_877 | p__Proteobacteria c__Gammaproteobacteria o__Xanthomonadales f__Sinobacteraceae |  | 6 |  |  |  |  |  |  |  |
| OTU_9 | p__Proteobacteria c__Alphaproteobacteria o__Rhizobiales f__Methylocystaceae |  | 1 | 2 |  |  |  |  |  |  |
| OTU_90 | p__Proteobacteria c__Alphaproteobacteria o__Caulobacterales f__Caulobacteraceae |  |  |  |  |  |  |  | 15 |  |
| OTU_917 | p__Chloroflexi c__Anaerolineae o__envOPS12 |  |  |  |  |  |  | 15 |  |  |
| OTU_980 | p__Verrucomicrobia c__[Spartobacteria] o__[Chthoniobacterales] f__[Chthoniobacteraceae] g__DA101 | 7 |  |  | 14 | 4 | 15 |  |  | 8 |
| OTU_99 | p__Proteobacteria c__Gammaproteobacteria o__Xanthomonadales f__Sinobacteraceae |  | 3 |  |  |  |  |  |  |  |

Table S3: pH thresholds for the soil health categories. Adapted from Hill and Sparling (2009). Values in column headers indicate the total number of sites that classified as that soil quality category.

| **Land use and Soil** | **Extremely low** | **Low** | **Healthy** | **High** | **Extremely High** |
| --- | --- | --- | --- | --- | --- |
|  | *n = 2* | *n = 42* | *n = 438* | *n = 35* | *n = 4* |
| Pastoral grasslands on non-organic soil | <5 | 5-5.49 | 5.5-6.3 | 6.31-6.6 | >6.6 |
| Pastoral grasslands on organic soil | <4.5 | 4.5-4.99 | 5-6.0 | 6.1-7.0 | >7.0 |
| Horticulture on non-organic soil | <5 | 5-5.49 | 5.5-7.2 | 7.3-7.6 | >7.6 |
| Horticulture on organic soil | <4.5 | 4.5-4.99 | 5-7.0 | 7.1-7.6 | >7.6 |
| Exotic forest on non-organic soils | <3.5 | 3.4-3.99 | 4-7.0 | 7.1-7.6 | >7.6 |
| Exotic forest on organic soils | excluded | | | | |

Table S4: Carbon (%) thresholds for the soil health categories. Adapted from Hill and Sparling (2009). Values in column headers indicate the total number of sites that classified as that soil quality category.

| **Soil type** | **Extremely low** | **Low** | **Healthy** |
| --- | --- | --- | --- |
|  | *n = 30* | *n = 61* | *n = 416* |
| Allophanic | <3 | 3-4.0 | >4 |
| Semi-arid, pallic and recent | <2 | 2-3.0 | >3 |
| Organic | excluded | | |
| All others | <2.5 | 2.5-3.5 | >3.5 |

Table S5: Total nitrogen (%) thresholds for the soil health categories. Adapted from Hill and Sparling (2009). Values in column headers indicate the total number of sites that classified as that soil quality category.

| **Land use** | **Extremely low** | **Low** | **Healthy** | **High** | **Extremely High** |
| --- | --- | --- | --- | --- | --- |
|  | *n = 8* | *n = 39* | *n = 228* | *n = 26* | *n = 83* |
| Pastoral grassland | <0.25 | 0.25-0.349 | 0.35-0.65 | 0.651-0.7 | >0.7 |
| Exotic | <0.10 | 0.10-0.199 | 0.20-0.60 | 0.61-0.7 | >0.7 |
| Horticulture | depends on the crops being grown – not assessed | | | | |

Table S6: Anaerobically mineralizable nitrogen (mg/kg) thresholds for the soil health categories. Adapted from Hill and Sparling (2009) with modifications as recommended by Mackay et al. (2013). Values in column headers indicate the total number of sites that classified as that soil quality category.

| **Land use** | **Extremely low** | **Low** | **Healthy** |
| --- | --- | --- | --- |
|  | *n = 10* | *n = 122* | *n = 388* |
| Pastoral grassland | <50 | 50-99.99 | ≥100 |
| Exotic | <20 | 20-39.99 | ≥40 |
| Horticulture | <20 | 20-99.99 | ≥100 |

Table S7: Olsen P (mg/kg) thresholds for the soil health categories. Adapted from Hill and Sparling (2009), with modifications as recommended by Oliver (2017). Values in column headers indicate the total number of sites that classified as that soil quality category.

| **Land use and Soil** | **Extremely low** | **Low** | **Healthy** | **High** |
| --- | --- | --- | --- | --- |
|  | *n = 84* | *n = 120* | *n = 272* | *n = 44* |
| Pastoral grassland on sedimentary and allophanic soils | <15 | 15-19.99 | 20-50 | >50 |
| Pastoral grassland on pumice and organic soils | <15 | 15-34.99 | 35-50 | >50 |
| Horticulture on sedimentry and allophanic soils | <20 | 20-49.99 | 50-50 | >50 |
| Horticulture on pumice and organic soils | <25 | 25-59.9 | 50-50 | >50 |
| Exotic on all soils | <5 | 5-9.99 | 10.0-500 | >50 |

Table S8: Macroporosity (% at -10kPa) thresholds for the soil health categories. Adapted from Hill and Sparling (2009). Values in column headers indicate the total number of sites that classified as that soil quality category.

| **Land use** | **Extremely low** | **Low** | **Healthy** | **Extremely High** |
| --- | --- | --- | --- | --- |
|  | *n = 99* | *n = 147* | *n = 248* | *n = 24* |
| Pastoral grassland and Horticulture | <6 | 6-9.99 | 10.0-30.0 | <30 |
| Exotic | <8 | 8-9.99 | 10.0-30.0 | <30 |

Table S9: Bulk density (t/m^3^) thresholds for the soil health categories. Adapted from Hill and Sparling (2009). Values in column headers indicate the total number of sites that classified as that soil quality category.

| **Soil type** | **Extremely low** | **Low** | **Healthy** | **High** | **Extremely High** |
| --- | --- | --- | --- | --- | --- |
|  | *n = 28* | *n = 46* | *n = 340* | *n = 84* | *n = 21* |
| Semi-arid, pallic and recent | <0.4 | 0.4-0.89 | 0.9-1.25 | 1.251-1.4 | >1.4 |
| Allophanic | <0.3 | 0.3-0.59 | 0.6-0.9 | 0.91-1.3 | >1.3 |
| Organic | <0.2 | 0.2-0.39 | 0.4-0.6 | 0.61-1.0 | >1.0 |
| All others | <0.7 | 0.7-0.79 | 0.8-1.2 | 1.21-1.4 | >1.4 |


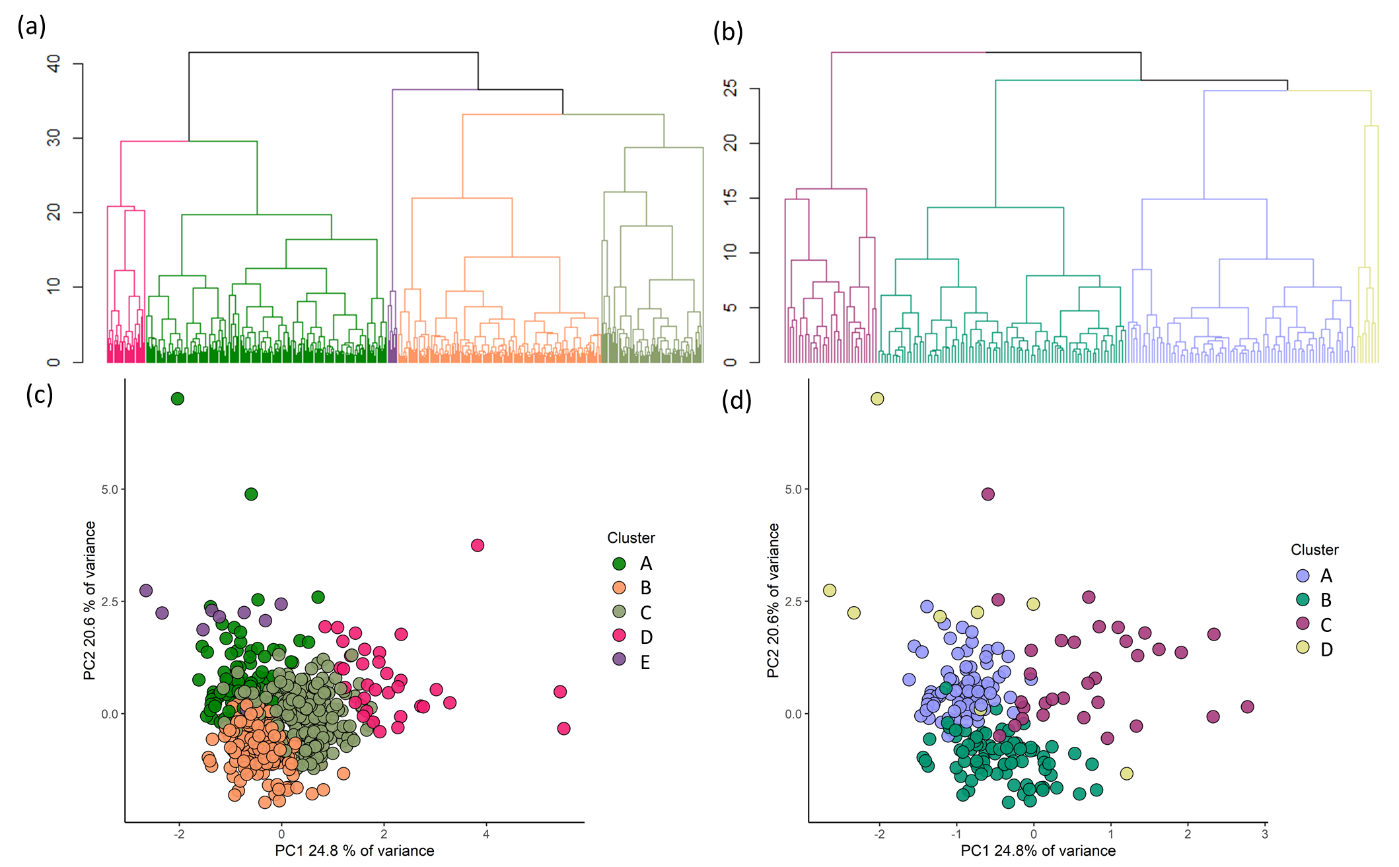


Figure S9: Ward clustering of the Euclidean distances in soil variables, and PCA showing the arrangements of clusters amongst (a, c) all managed sites and (b, d) non-pastoral grassland managed sites.

**References:**

Hill RB, Sparling GP (2009) Soil quality monitoring. Land and soil monitoring: A guide for SoE and regional council reporting. Land Monitoring Forum, NZ

Mackay A, Dominati E, Taylor M (2013) Soil quality indicators: The next generation. report prepared for land monitoring forum of regional councils. Client report number: RE500/2012/025

Oliver M (2017) Soil quality in the Marlborough region in 2016. MDC Technical Report No: 17-003
